# Pet Flea and Tick Control Exposure During Pregnancy and Early Life Associated with Decreased Cognitive and Adaptive Behaviors in Children with Developmental Delay and Autism Spectrum Disorder

**DOI:** 10.3390/ijerph22071149

**Published:** 2025-07-19

**Authors:** Amanda J. Goodrich, Daniel J. Tancredi, Yunin J. Ludeña, Ekaterina Roudneva, Rebecca J. Schmidt, Irva Hertz-Picciotto, Deborah H. Bennett

**Affiliations:** 1 Department of Public Health Sciences, School of Medicine, University of California Davis, Davis, CA 95616, USA; 2 Department of Pediatrics, University of California Davis, Sacramento, CA 95817, USA; 3 Medical Investigation of Neurodevelopmental Disorders (MIND) Institute, University of California Davis, Sacramento, CA 95817, USA

**Keywords:** insecticides, pesticides, flea and tick, neurodevelopmental disorders, pregnancy, early life

## Abstract

Approximately 18% of U.S. children experience cognitive and behavioral challenges, with both genetic and environmental contributors. We examined if household insecticides, particularly those used in and around the home and on pets, are associated with neurodevelopmental changes. Data were from children aged 24–60 months in the CHARGE study with the following classifications: autism spectrum disorder (ASD, *n* = 810), developmental delay (DD, *n* = 192), and typical development (TD, *n* = 531). Exposure to indoor, outdoor, and pet insecticides was reported for the period from three months pre-conception to the second birthday. Cognitive and adaptive functioning were assessed using the Mullen Scales of Early Learning and Vineland Adaptive Behavior Scales. Linear regression was used to evaluate associations by diagnostic group, adjusting for confounders. Flea/tick soaps, shampoos, and powders used during year two were significantly associated with lower cognitive and adaptive scores in children with ASD after FDR correction. Flea/tick skin treatments in early pregnancy were associated with reduced scores in the DD group, though not significant after correction, especially when used with high frequency. No associations were observed in TD children. These findings underscore the need to examine early-life exposure to non-agricultural insecticides as modifiable risk factors for neurodevelopment.

## 1. Introduction

As many as one in five children in the United States experience deficits in cognitive, adaptive, communicative, and psychomotor abilities [1,2]. While genetic factors appear to play a substantial role in these deficits, the growing literature has identified that environmental factors, e.g., chemical, biological, and nutritional exposures, may significantly contribute to their etiology [3,4,5,6,7].

Insecticides, a diverse group of chemicals often designed to disrupt the neurological systems of pests, pose potential risks to human neurodevelopment. Household insecticide exposure, in particular, has emerged as a suspected contributor to neurodevelopmental toxicity during early life. Before the early 2000s, organophosphorus (OP) compounds were the predominant active ingredients in household insecticide sprays, after the banning of organochlorines in household insecticides in the United States during the 1970s and 1980s. Notably, OP insecticides such as chlorpyrifos and diazinon were extensively used in residential settings until they were banned by the United States Environmental Protection Agency (EPA) from household products, with phased eliminations occurring in 2001 for chlorpyrifos and in 2002 and 2004 for indoor and outdoor formulations of diazinon, respectively [8,9]. Following this phase-out, pyrethroids became the main active compounds in household insecticides [10].

Epidemiological studies have reported associations between both OP and pyrethroid exposures and neurodevelopmental outcomes, including autism spectrum disorder (ASD) [11,12,13,14,15,16,17,18,19,20,21]. While most of these studies have focused on proximity to agricultural pesticide applications [12,13,19] or short-lived biomarkers measured at specific time points during pregnancy or early childhood [14,22,23,24], our group recently found an association between caregiver reports of professionally and non-professionally applied household insecticides, especially repeated exposures during pregnancy and early life, and ASD in the CHARGE study [21]. While the neurodevelopmental risks associated with OP exposure are well-documented, the evidence regarding pyrethroid exposure is less conclusive.

Pet flea and tick control products are widely used in the United States, where an estimated 70% of households own pets, with dogs and cats being the most common [25]. To manage flea and tick infestations, a variety of chemical treatments are employed, including topical spot-on products, collars, oral medications, and soaps/shampoos, many of which contain neuroactive compounds such as pyrethroids, neonicotinoids, fipronil, and insect growth regulators [26]. These products are designed to disrupt the nervous systems or developmental cycles of fleas and ticks; however, little is known about their neurodevelopmental effects in humans, particularly during critical windows of development. 

This study leverages extensive caregiver-reported data on household insecticide use from the CHARGE (Childhood Autism Risk from Genetics and the Environment) study, detailing the timing and frequency of insecticide use from three months before conception through the first two years of a child’s life. We aimed to examine potential impacts of these exposures on cognitive and adaptive behaviors in children, utilizing standardized composite and subscale scores from the Mullen Scales for Early Learning (MSEL) [27] and Vineland Adaptive Behavior Scales (VABS) [28]. Analyses were conducted separately for children with ASD, developmental delay without ASD (DD), and typical development (TD) classifications.

## 2. Materials and Methods

### 2.1. Study Population

The study sample was drawn from the CHARGE study, a population-based case–control study initiated in 2003 to investigate environmental and genetic risk factors for autism and other neurodevelopmental disorders, along with gene-by-environment interactions [29]. Eligible participants were recruited into CHARGE from within California and included children aged 24 to 60 months at the time of recruitment, born in California, and currently residing with at least one biologically related parent who was proficient in English or Spanish. Children who either had ASD or DD were identified through the California Department of Developmental Services (DDS), which coordinates services to children with developmental disabilities through their system of Regional Centers (RCs). Families of children with ASD were also recruited from other UC Davis MIND Institute studies or from people who contacted the study team directly. General population (GP) controls were selected through the California State Vital Statistics birth files and frequency-matched to ASD cases based on age, sex assigned at birth, and RC catchment area distribution. Subjects were excluded from analyses if the questionnaire on insecticide exposures was not completed or the child had a known genetic cause for their DD diagnosis (e.g., Down syndrome, Fragile X syndrome, etc.).

Subjects were eligible for this study if they were in the CHARGE study and classified as ASD, DD. or TD (see classification criteria below). Subjects were excluded if they did not complete the questionnaire on insecticide exposures or had a known genetic cause for their condition. The study was approved by institutional review boards of the University of California. Written informed consent was obtained from all parents or legal guardians prior to collection of any data.

### 2.2. Diagnostic and Outcome Measurements

Upon enrollment, most children were assessed at the UC Davis MIND Institute to confirm diagnoses; however, a small percentage were seen at the UCLA Neuropsychiatric Institute. Children with previous ASD diagnoses were assessed using the Autism Diagnostic Observation Schedules-2 (ADOS-2) [30,31] and the Autism Diagnostic Interview–Revised (ADI-R) [32,33,34]. Children without a prior ASD diagnosis were screened for ASD symptoms using either the Social Communication Questionnaire (SCQ) [35] or clinician judgment. If the total SCQ score was 15 or above [36], or if the clinician suspected ASD, assessments with the ADI-R and ADOS-2 were conducted during a second visit. Cognitive function was evaluated in all children using the Mullen Scales of Early Learning (MSEL), and adaptive skills were assessed by administering the Vineland Adaptive Behavior Scales (VABS) to the primary caregiver. Children who did not meet the CHARGE criteria for ASD on both the ADI-R and ADOS-2 were further classified based solely on their MSEL and VABS composite scores. Those scoring below 70 on either measure were classified as having DD, while those scoring 70 or above on both were classified as TD.

The MSEL is a clinician-administered tool designed to evaluate cognitive functioning in young children across multiple domains. It generates an Early Learning Composite score based on performance in four subscales: receptive language, expressive language, visual reception, and fine motor skills. Standard scores for the MSEL subscales are normalized against age-based norms, with a mean ± SD score of 100 ± 15. To mitigate the potential for floor effects in children with significant developmental delays, developmental quotients (DQ) were calculated. DQs were derived by dividing the age-equivalent score obtained from each subscale by the child’s chronological age and multiplying by 100, with higher DQs indicating better cognitive performance.

Adaptive functioning was measured using the VABS, a semi-structured interview administered to caregivers, designed to assess daily adaptive behaviors necessary for personal and social sufficiency. The VABS provides an Adaptive Behavior Composite score, alongside subscale scores in the domains of communication, daily living skills, socialization, and motor skills. Similarly to the MSEL, scores provided are standardized scores with a mean ± SD of 100 ± 15. DQs are used and calculated the same way as with MSEL. Higher DQs reflect greater adaptive functioning.

### 2.3. Household Insecticide Exposure

We determined prenatal and early life exposure to indoor, outdoor, and flea and tick control insecticides, as described elsewhere [21]. In brief, the primary caregiver (usually the mother) was asked during a comprehensive interview about the use of insecticides in the home, who applied them (which parent, others in the household, or professional applicators), where they were applied (indoor, outdoor, on pets), during which month and/or year of the index period (beginning 3 months before pregnancy through the second year of life) they were applied, and how frequently they were applied during that period. The questionnaire was modified partway through the study, with changes that included categorizing professionally and non-professionally applied insecticide products into indoor vs. outdoor use at the time of data collection, rather than collecting free text descriptions of application location and manually categorizing them after data entry. Additionally, in the later version of the questionnaire, separate questions were included for each type of pet flea/tick product, while in the earlier version, one question grouped pet sprays, powders, and skin-applied treatments together, and responses were manually categorized to distinguish between skin-applied treatments and other forms (e.g., sprays, powders). Product text fields were manually reviewed to exclude items that did not contain insecticides, and brand names were cross-referenced against the EPA’s Insecticide Product and Label System (https://ordspub.epa.gov/ords/insecticides/f?p=PPLS:1 (accessed on 15 December 2022)) to verify the inclusion of an active insecticidal compound. Products that contained borax, such as poisoned bait containers, were excluded from analyses, as their minimal insecticide surface area is unlikely to produce significant volatilization or meaningful exposure.

We categorized exposure types into several groups: professionally applied indoor insecticides, non-professionally applied indoor insecticides, any indoor insecticide application, non-professionally applied outdoor insecticides, any outdoor insecticide application, and pet flea and tick treatments including collar use, skin application, a combined category for soap, shampoo, and powder use, and any flea and tick treatment. Exposure frequency was classified into three categories for the pre-pregnancy period and each trimester: no exposure, exposure for 1–2 months, and exposure for all 3 months. Similarly, for the entire pregnancy period, exposure frequency was grouped into no exposure, exposure for 1–5 months, and exposure for 6–9 months. Exposure frequency was not asked for the postnatal period.

### 2.4. Confounders

To illustrate the hypothesized causal pathways among key study variables, a Directed Acyclic Graph (DAG) was constructed using the Dagitty web platform [37]. The DAG was used to identify a minimally sufficient set of covariates to control confounding in the relationship between household insecticide use and child cognitive and adaptive functioning (Figure S1, Supplementary Materials). Based on this framework, the final adjustment set included: year of conception (modeled as a cubic polynomial), maternal education (bachelor’s degree and higher vs. some college or less), maternal race/ethnicity (non-Hispanic white (NHW) vs. non-NHW), home ownership at time of enrollment (yes/no), season of conception (Nov–Feb, Mar–Jun, and Jul–Oct), urbanicity (mother’s address at time of delivery within an area determined by U.S. Census classification closest to the child’s birth year: 2000, 2010, or 2020; yes/no), and maternal mental health problems during pregnancy (yes/no). Because data on maternal mental health were not collected prior to October 2005, this variable was missing for 382 participants. To preserve sample size, we used multiple imputation to estimate missing values, following a procedure described previously [21]. Briefly, we performed 50 imputations using a Markov Chain Monte Carlo method under the assumption of a multivariate normal distribution. Including maternal mental health in the fully adjusted models did not alter the effect estimates. Therefore, to maximize statistical power, this variable was omitted from the final analytic models.

Covariate data obtained from parent interviews were validated with birth and medical records, as described previously [21]. The child’s birth address was classified as urban vs/non-urban using Census definitions and based on parent-reported addresses at birth in conjunction with Census population data.

### 2.5. Statistical Analyses

Univariate analyses were used to assess exposure and covariate distributions. All variables presented in Table 1 were either collected directly or derived from study interview responses. We estimated betas and 95% confidence intervals (CIs) for associations between each of the various application types of household insecticides for each time period, separately, and the composite DQs for MSEL or VABS fitting multiple linear regression models adjusted for confounders, among each of the diagnostic groups (ASD, DD, TD). Considering the differences in diagnosis rates by the child’s sex, we assessed effect modification by sex by including the product of each exposure by the child’s sex. A *p*-value < 0.10 for the multiplicative term was considered significant evidence for an interaction. In secondary analyses, we examined the associations between each of the application types of household insecticides and the different subscales for the MSEL and VABS using separate multiple linear regressions, adjusted for the same confounders.

To address the issue of multiple hypothesis testing, we utilized the false discovery rate (FDR) method in our primary analyses, which involved examining the relationship between each type of insecticide application (yes/no) and either MSEL or VABS DQ. The Benjamini–Hochberg procedure was employed to control the proportion of false positives among the significant findings, thereby enhancing the reliability of our results across independent comparisons. Associations with a Q-value after FDR correction (QFDR) of less than 0.05 were considered statistically significant, while those with a QFDR less than 0.10 were deemed borderline significant. Stata (SE version 18.0; StataCorp, College Station, TX, USA) was used for all analyses.

## 3. Results

### 3.1. Participant Characteristics

At the time of this study, *n* = 1772 participants had been enrolled and classified as ASD (*n* = 875), DD (*n* = 343) or TD (*n* = 554). These included *n* = 599 recruited from GP (final study classification of these were: *n* = 7 ASD, 38 DD, 554 TD), with the remaining *n* = 1173 children recruited from DDS, from other MIND studies, or from people who contacted the study team directly. Of these, *n* = 239 subjects were excluded from analyses for having a known genetic cause for their DD diagnosis (*n* = 137) or not completing the questionnaire on insecticide exposures (*n* = 65 ASD, *n* = 14 DD, *n* = 23 TD). Of the *n* = 1533 remaining, *n* = 810 were classified as ASD, *n* = 192 as DD, and *n* = 531 as TD. A further *n* = 20 ASD, *n* = 7 DD, and *n* = 11 TD were missing covariates.

Enrolled children were conceived between 1997 and 2018, with TD children born in slightly later years because enrollment in the first few years for TD children was delayed due to a lengthy process for obtaining access to the State’s birth files (Table 1). The children in our study were ethnically diverse, with 47% non-Hispanic white (derived from parent-reported parental race/ethnicity), 32% Hispanic, and 21% other races. Due to the high male/female ratio among those with ASD and our frequency matching of GP recruitment with ASD, participating children were predominantly male (81%).

### 3.2. Distribution of MSEL and VABS DQs

Distributions (mean ± SD) of developmental quotients of MSEL and VABS composite and subscale DQs are presented in Table S1. All mean DQs were roughly similar for ASD and DD, except for MSEL Fine Motor scores and VABS Motor Skills, which were higher for ASD than for DD, and as expected, VABS Socialization which was lower for ASD than DD. TD DQs were 45–55 points higher than either ASD or DD.

### 3.3. Distribution of Insecticide Exposure

In our study population, the most frequently reported insecticide application was any indoor insecticide (44.2%), followed by any outdoor insecticide (40.0%; Table S2 The least commonly reported exposure was from flea and tick collars (6.3%), with lower frequencies also observed for indoor insecticides applied by a professional (11.8%) and flea and tick shampoos, powders, or soaps (14.9%). For all insecticide types other than flea and tick collars, reported use was lower during pregnancy compared to the child’s first and second years of life, with indoor and outdoor use approximately doubling in the postnatal period. Among prenatal exposures—excluding flea and tick products—use was most frequently reported during the second trimester relative to the first and third trimesters.

### 3.4. Insecticide Exposure and Neurodevelopmental Skills

**Figure 1:**
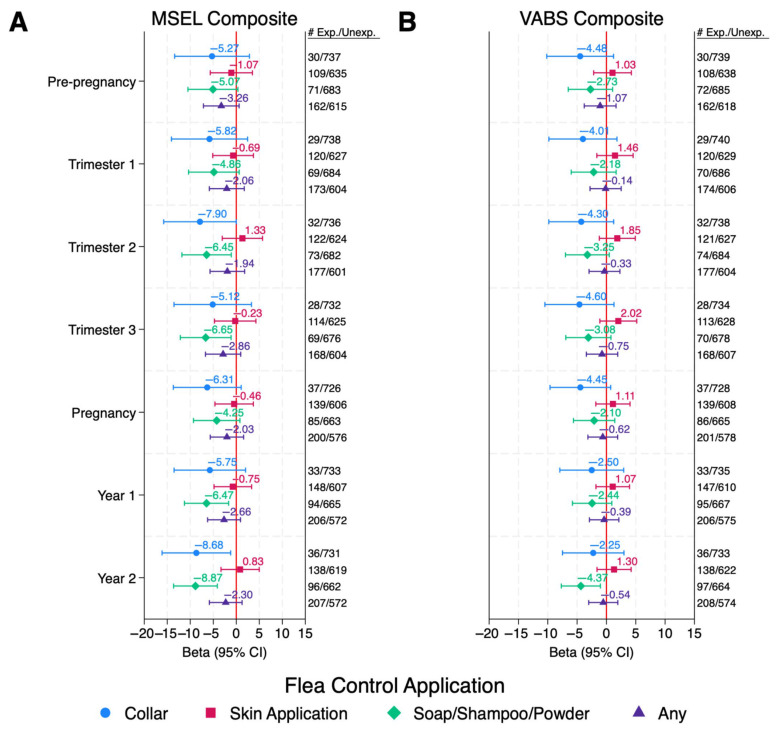
ASD Children: Associations Between Pet Flea Control Exposures and Cognitive and Adaptive Behaviors Among Children with ASD. Values are adjusted betas and 95% confidence intervals (bars) from linear regression, modeled separately for each period and each exposure. Outcomes are Composite DQ scores for either (**A**) MSEL or (**B**) VABS. Numbers exposed and unexposed for each exposure/time period are listed to the right of the figure. Models are adjusted for child’s year of conception (cubic), mother’s education (bachelor’s degree or higher vs. less than bachelor’s degree), mother’s race/ethnicity (non-Hispanic White vs. other), home ownership at time of enrollment (yes/no), mother’s address in urban area at time of delivery (yes/no), season of conception (Nov–Feb, Mar–Jun, and Jul–Oct), and child’s age at time of assessment (months). Abbreviations: ASD, autism spectrum disorder; Exp, Exposed; MSEL, Mullen Scales of Early Learning; Unexp, Unexposed; VABS, Vineland Adaptive Behavior Scales.

Of the flea and tick applications, reported use in the ASD group of soaps, shampoos, or powders or collars during any point in the index period was associated with MSEL Composite DQs 4–9 points lower than those that were not exposed (Figure 1A and Table S3). These associations reached statistical significance from mid-pregnancy (2nd trimester) through the 2nd year of life for soaps, shampoos, and powders, and in the second trimester and second year of life for collars; however, after FDR correction, only soaps, shampoos, and powders exposure during the second year of life remained marginally significant (QFDR = 0.09).

**Figure 2:**
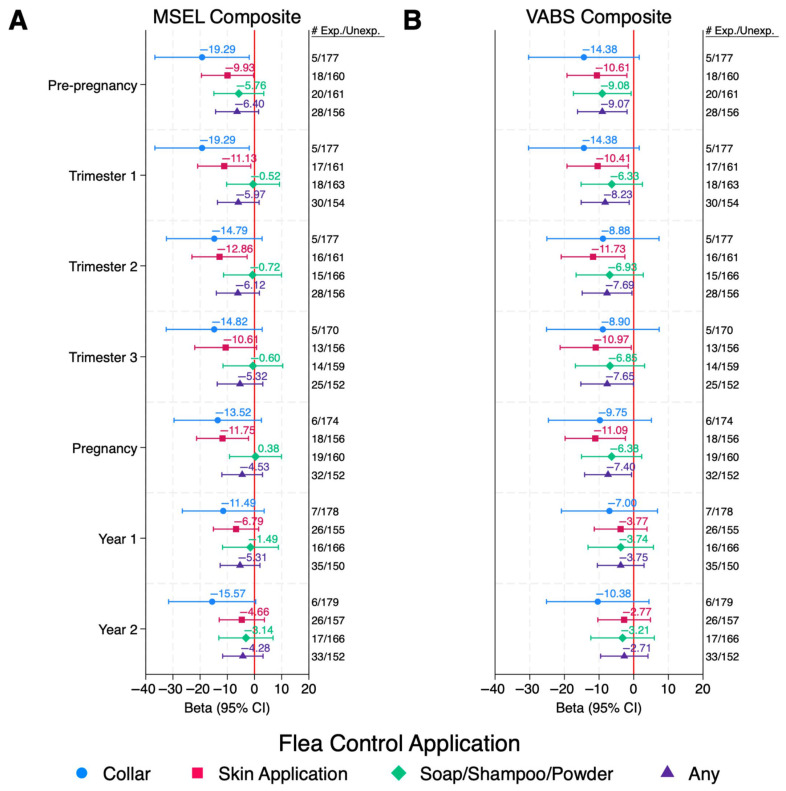
DD Children: Associations Between Pet Flea Control Exposures and Cognitive and Adaptive Behaviors Among Children with DD. Values are adjusted betas and 95% confidence intervals (bars) from linear regression, modeled separately for each period and each exposure. Outcomes are Composite DQ scores for either (**A**) MSEL or (**B**) VABS. Numbers exposed and unexposed for each exposure/time period are listed to the right of the figure. Models are adjusted for child’s year of conception (cubic), mother’s education (bachelor’s degree or higher vs. less than bachelor’s degree), mother’s race/ethnicity (non-Hispanic White vs. other), home ownership at time of enrollment (yes/no), mother’s address in urban area at time of delivery (yes/no), season of conception (Nov–Feb, Mar–Jun, and Jul–Oct), and child’s age at time of assessment (months). Abbreviations: ASD, autism spectrum disorder; Exp, Exposed; MSEL, Mullen Scales of Early Learning; Unexp, Unexposed; VABS, Vineland Adaptive Behavior Scales.

Reported use of skin applications of flea and tick control products in the DD group during any point in the pre-pregnancy or pregnancy periods were also associated with lower cognition with MSEL Composite DQs 10–13 points lower than those that were not exposed, which represents a loss of two thirds the SD or more for those exposed (Figure 2A and Table S3). While exposures during all periods except trimester 3 were statistically significant, the significance did not hold after FDR correction for multiple comparisons (QFDR = 0.66–0.86). Similar associations were observed with VABS DQs.

Reported use of any flea and tick control product during pre-pregnancy and pregnancy in the DD group was associated with lower adaptive behaviors, with VABS Composite DQs 8–9 points lower than those that were not exposed; however, the significance did not hold after FDR correction for multiple comparisons (QFDR = 0.83–0.94). Reported use of flea and tick collars during pre-pregnancy and trimester 1 were statistically significantly associated with lower cognition with MSEL Composite DQs 19 points lower than those that were not exposed; however, the confidence intervals were very large due to the small number of subjects exposed, and the significance did not hold after FDR correction (QFDR = 0.71–0.99).

Among children with ASD or TD, inverse or null associations were observed with all applications of reported indoor or outdoor insecticide exposure during the index period and MSEL Composite DQs (Table S3). Among DD children, indoor applications during the pre-pregnancy and pregnancy periods were associated with decreased MSEL DQs; however, these associations did not reach statistical significance.

No associations were observed among TD children for any insecticide application in any period (Table S3). Reported use of indoor professionally applied insecticides in the DD group could not be assessed due to small sample sizes. Similar, or slightly attenuated, results were observed for VABS composite DQs in all diagnostic groups (Figure 1B and Figure 2B and Table S4).

Effect modification by child’s sex was observed in the ASD group for exposure to flea/tick soaps, shampoos, and powders in all time periods, flea/tick skin applications in pre-pregnancy and trimester 1, any flea/tick control product pre-pregnancy, trimester 1, pregnancy average, and year 1 on MSEL Composite DQs, with females having stronger negative effects of each insecticide product than males (*p* for interaction < 0.10; Table S5). Effect modification by child’s sex was also observed in the ASD group for exposure to any outdoor insecticides in trimesters 2 and 3, and year 2, with non-significant negative effects observed in females, and null effects observed in males. While the associations in females did not reach statistical significance, the sample size for females was, by design, much lower than that of males. Among the TD children, effect modification by child’s sex was observed for exposure to indoor and outdoor insecticides (non-professionally applied and any application) in various time points and MSEL Composite DQs, with males generally having stronger negative effects of each insecticide than females. Effect modification by sex was not observed for the DD group, except with exposure to any outdoor insecticide in year 1, with stronger negatives effects observed in males. Similar results were observed with VABS Composite DQs (Table S6). Effect modification by sex for flea/tick collars and indoor professionally applied insecticides among female children in all diagnostic groups, as well as outdoor non-professionally applied insecticide and flea and tick skin applications in the DD group, could not be evaluated due to small sample sizes of females.

Associations between each reported insecticide application and the subscales of MSEL were similar to the Composite scores for each diagnostic group (Table S7). Associations with reported flea and tick collar use in year 2 among ASD children were stronger and reached statistical significance with Expressive Language and Visual Receptivity (Beta (95% CI): −10.60 (−18.90, −2.30) and −8.94 (−17.40, −0.48), respectively). Exposure during trimester 2 or the whole pregnancy period was associated with a statistically significant 8-point (nearly an SD) lower VABS Communication DQ, and an 8-point lower VABS Fine Motor Skills DQ in trimester 3 (Table S8). Associations with reported flea/tick soap, shampoo, and powder use during any time period among ASD children were strongest with Receptive Language, with estimated mean scores 6–10.5 points lower among those exposed than those not exposed during the same time period, and in year 2, these associations reached statistical significance with every MSEL subscale as well as VABS Communication and Daily Living Skills subscales (Tables S7 and S8).

DD children with reported exposure to flea and tick skin applications during pre-pregnancy, any pregnancy period, or the first year of life was statistically significantly associated with a decrease of 12–18 points (well over 1 SD difference) in MSEL Visual Receptivity DQs and 10–19 points (nearly 2 SD’s) in VABS Socialization DQs, compared to those that were not exposed (Tables S7 and S8). MSEL Receptive Language DQs were also statistically significantly 10–14 points lower when DD children were exposed during pre-pregnancy or any pregnancy period compared to those that were not exposed.

High frequency of exposures (every month for trimesters or at least 6 months out of the entire pregnancy period) of non-professionally applied indoor insecticides in every time period in the DD group was associated with a statistically significant decrease in MSEL Composite DQs of 10–12.5 points (Table S9). Associations were attenuated or null with less frequent use. While there was a similar trend towards decreasing VABS Composite DQs in these same time periods, none of these associations reached statistical significance (Table S10). In the ASD and TD groups, associations were mostly null, or slightly inverse, with similar effect estimates observed in both frequency groups.

## 4. Discussion

This study builds upon the growing body of evidence that household insecticide exposure, including pet flea and tick control products and indoor spray insecticides, can adversely affect neurodevelopmental outcomes in children. By examining exposure across distinct time windows and application types, this analysis highlights nuanced associations between household insecticide use and cognitive and adaptive functioning, as measured by MSEL and VABS.

The association between pet flea and tick control products and lower cognitive and adaptive functioning scores, particularly in children with ASD, aligns with prior studies documenting the neurotoxic potential of insecticides [38]. The consistent significance, in children with ASD, of exposures to flea and tick soaps, shampoos, and powders during the second year of life, when a child interacts more with their environment and potentially with pets in the home, underscores the importance of examining chronic, low-level exposures during sensitive developmental periods. Similarly, the observed associations in DD children of exposure to flea and tick skin applications during pre-pregnancy and early pregnancy suggest that the timing of exposure is critical. Other possible reasons for the association in early childhood only occurring in the ASD group are that children with ASD are also known to bond more readily with their pets than with other humans [39,40] and may have increased hand-to-mouth behaviors [41,42], with both factors leading to a higher likelihood of ingesting flea/tick insecticides after touching their pets.

Associations between non-professionally applied indoor insecticides and lower neurodevelopmental scores, particularly cognitive measures, in DD children were only observed when the exposure occurred every month of a trimester or at least 6 months of the full pregnancy period, indicating higher and/or more consistent exposures. The lack of associations for fewer months of exposure may reflect the small sample size of DD children. Given the magnitude of the observed associations with frequent exposures, further investigation is warranted. The stronger associations observed with indoor applications is potentially driven by higher insecticide concentrations, given that indoor insecticides linger longer than outdoor ones, the latter being degraded by sunlight and dispersed more readily with wind [43,44]. Very few DD children were exposed to professionally applied insecticides—too few for analysis (see Table S2).

The findings align with established biological pathways for insecticide-induced neurotoxicity. Neonicotinoids, including imidacloprid and dinotefuran, are a class of insecticides commonly used in pet flea and tick control products, which act as agonists of nicotinic acetylcholine receptors (nAChRs) in the central nervous system [45]. Exposure to low concentrations of these compounds has been shown to stimulate the extracellular signal-regulated kinase (ERK/MAPK) signaling pathway through activation of nAChRs and subsequent intracellular calcium release [46]. During critical periods of neurodevelopment, such as the second year of life, this over-stimulation can disrupt the balance of excitatory and inhibitory signaling, impairing synaptic plasticity and leading to long-term deficits in cognitive and behavioral function [47,48]. Additionally, imidacloprid exposure has been shown to induce oxidative stress and mitochondrial dysfunction [49,50], both of which are linked to neuroinflammation and neuronal injury. Emerging evidence suggests that these processes may contribute to alterations in synaptic connectivity, a hallmark feature of neurodevelopmental disorders [51].

Fipronil, another common active ingredient in pet flea and tick control products, primarily acts as a gamma-aminobutyric acid (GABA) receptor antagonist, impairing inhibitory neurotransmission [52]. This disruption can lead to hyperexcitation in neural networks, potentially affecting the maturation of neural circuits critical for cognitive and behavioral development. Fipronil exposure has also been associated with oxidative stress [53,54] and endocrine disruption [54,55], both of which are relevant to neurodevelopmental outcomes [51,56]. Importantly, its lipophilic nature allows it to accumulate in fatty tissues, including the brain [54], potentially prolonging its effects during critical developmental windows.

### Strengths and Limitations

While this study provides valuable insights into the neurotoxic effects of household insecticides, several limitations must be acknowledged. As with any self-reported exposure data, the responses of the primary caregivers to questions on insecticide use may have been subject to recall bias, if caregivers of children with developmental delays or ASD report differentially as compared with those caring for TD children. This could lead to overreporting of exposures perceived as harmful in those with affected children or under-reporting by those with unaffected children—in both cases, leading to differential misclassification that would tend to inflate associations. However, one study [57], aimed at ascertaining if self-report of multiple exposures during pregnancy was random or systematic, found that reporting errors of most exposures (75%) were random, with the authors concluding that recall bias is exposure-specific. If these recall errors were systematic, the potential exists that they are exaggerated away from the null. The reliance on self-reported exposure histories also raises concerns about the accuracy of timing and frequency of insecticide applications, especially over extended recall periods. However, these types of errors tend to be non-differential, biasing the results toward the null.

We were also unable to ascertain specific insecticide formulations or active ingredients, thereby limiting the capacity to determine the relative toxicity of a given ingredient. Although participants were asked to provide specific brand names of insecticides used, detailed recall was often limited. Many respondents gave general brand names such as “Raid” without specifying the exact product type (e.g., “Raid Max Ant and Roach Killer” vs. “Raid Wasp & Hornet Killer”), which can differ in active ingredients. Additionally, the timing of product purchase was not collected, preventing identification of the exact formulation in use. As a result, accurately determining the specific insecticidal compounds to which participants were exposed was challenging.

Additionally, insecticide residues in food and exposures outside the home environment, such as in workplaces or childcare settings, were not assessed. These settings often use insecticides as part of routine maintenance, which likely contributed to additional exposures not reflected in our data. As a result, total insecticide exposure may have been underestimated. We also had very few people in the DD group who were exposed to certain insecticides, resulting in models that were underpowered, or unable to be assessed.

Residual confounding is another concern, particularly with respect to socioeconomic status (SES), which may influence both insecticide use patterns and neurodevelopmental outcomes. Despite efforts to adjust for SES-related variables, such as maternal education and homeownership, more nuanced SES factors may not have been fully accounted for. Families using pesticides may experience higher pest burdens due to environmental factors, such as older housing or poor maintenance, which could independently contribute to stressors or health risks that affect neurodevelopment. Conversely, those with higher SES might be more likely to have their homes professionally sprayed on a regular basis. This dynamic of structural factors may result in residual confounding that biases the results in unpredictable directions and complicates the interpretation of causality.

Lastly, we observed stronger associations when exposures to pet flea and tick control products occurred in early life compared to during pre-pregnancy or pregnancy. It is possible that the recall for early life exposures is more accurate due to it occurring closer to the time in which the exposures were reported. Given that the analyses were performed separately within diagnosis classification, there would be no recall bias. Therefore, if recall error is occurring in the earlier time periods, this would likely be non-differential to the outcome, biasing our results toward the null.

This study has several notable strengths. The use of detailed, self-reported data on the timing, type, and frequency of pesticide exposures provides valuable granularity, allowing for the examination of specific exposure windows critical for neurodevelopment. The large sample size, which includes children with and without ASD, enhances the statistical power and generalizability of findings. Additionally, the rigorous diagnostic classification of ASD using gold-standard tools, such as the ADOS-2 and ADI-R by trained clinicians who have attained reliability on the instruments they administer, ensures reliable identification of cases and minimizes the potential for outcome misclassification bias. The study’s diverse population, encompassing a broad range of socioeconomic, demographic, racial, ethnic and cultural backgrounds, further supports the relevance of findings to varied community settings.

## 5. Conclusions

These findings from the CHARGE study contribute to the growing body of evidence suggesting that household insecticide use, particularly indoor insecticides and products used for flea and tick control, may negatively affect the development of critical domains, namely cognitive/intellectual and adaptive/functional living skills, especially in children with ASD, but also for those with other neurodevelopmental impairments. Future research should aim to replicate these timing-specific associations, evaluate exposure frequency and intensity, and assess cumulative insecticide exposures from diverse sources, including dietary intake, occupational and childcare environments, and residential proximity to treated areas, to more fully elucidate their potential impact on neurodevelopment.

## Figures and Tables

**Table 1:** Subject Characteristics by CHARGE Study Diagnostic Group.

	ASD (*n* = 810)	DD (*n* = 192)	TD (*n* = 531)
Male Sex (%)	677 (83.6%)	136 (70.8%)	432 (81.4%)
Child’s Race/Ethnicity ^A^			
Non-Hispanic White	381 (47.0%)	65 (34.2%)	271 (51.3%)
Hispanic	254 (31.4%)	91 (47.9%)	148 (28.0%)
Other	175 (21.6%)	34 (17.9%)	109 (20.6%)
Year of Conception	2004 (2000, 2009)	2004 (2002, 2007)	2004 (2002, 2007)
Season of Conception			
Nov–Feb	268 (33.6%)	60 (31.7%)	165 (31.7%)
Mar–Jun	267 (33.5%)	64 (33.9%)	185 (35.5%)
Jul–Oct	262 (32.9%)	65 (34.4%)	171 (32.8%)
Child’s Age at Assessment (months)	47.0 (39.0, 54.0)	47.0 (42.0, 54.0)	44.0 (35.0, 52.0)
Mother’s Race/Ethnicity ^B^			
Non-Hispanic White	451 (56.0%)	84 (43.8%)	340 (64.6%)
Hispanic	199 (24.7%)	74 (38.5%)	105 (20.0%)
Other	156 (19.4%)	34 (17.7%)	81 (15.4%)
Mother’s Age at Conception (yrs) ^C^	30.0 (26.0, 34.0)	28.0 (24.0, 33.0)	30.0 (26.0, 34.0)
≥35 yrs (% yes)	174 (21.8%)	36 (19.0%)	104 (20.0%)
Father’s Age at Conception (yrs) ^D^	32.0 (28.0, 37.0)	31.0 (25.0, 37.0)	32.0 (28.0, 36.0)
≥35 yrs (% yes)	271 (34.5%)	62 (33.9%)	179 (34.6%)
Maternal Pre-pregnancy BMI ^E^	24.5 (21.8, 29.2)	25.2 (21.9, 29.7)	24.2 (21.6, 28.2)
Underweight	31 (3.9%)	4 (2.1%)	15 (2.8%)
Normal	406 (50.7%)	84 (44.9%)	286 (54.3%)
Overweight	181 (22.6%)	56 (29.9%)	137 (26.0%)
Obese	183 (22.8%)	43 (23.0%)	89 (16.9%)
Gestational Age < 37 weeks (% yes) ^F^	83 (10.5%)	32 (16.9%)	48 (9.2%)
Parity ^G^			
1	399 (49.4%)	68 (35.4%)	211 (40.0%)
2	282 (34.9%)	67 (34.9%)	203 (38.4%)
3+	126 (15.6%)	57 (29.7%)	114 (21.6%)
Gestational Diabetes (% yes) ^H^	78 (9.7%)	20 (10.5%)	30 (5.7%)
Had any mental health condition before or during pregnancy (% yes) ^I^	247 (42.7%)	54 (33.3%)	101 (22.3%)
Mother Born in US (% yes) ^J^	600 (74.6%)	141 (73.4%)	441 (83.2%)
Private Health Insurance ^K^	629 (78.8%)	115 (60.5%)	442 (84.5%)
Home Ownership (% yes) ^L^	512 (63.7%)	103 (54.5%)	396 (75.1%)
Financial Hardship (%yes) ^M^	168 (20.8%)	54 (28.6%)	80 (15.1%)
Mother’s Education			
Some College	122 (15.1%)	64 (33.3%)	69 (13.0%)
AA or Technical Degree	325 (40.1%)	73 (38.0%)	177 (33.3%)
Bachelor’s Degree	242 (29.9%)	42 (21.9%)	197 (37.1%)
Graduate Degree or higher	121 (14.9%)	13 (6.8%)	88 (16.6%)
Lived in single-family home			
Pre-pregnancy ^N^	504 (66.8%)	119 (68.0%)	382 (75.8%)
Entire Pregnancy ^O^	486 (63.6%)	109 (60.6%)	380 (73.9%)
Year 1 ^P^	505 (65.8%)	108 (60.0%)	396 (76.9%)
Year 2 ^Q^	529 (69.2%)	113 (63.1%)	412 (80.2%)
Child’s birth address in urban area ^R^	736 (92.0%)	171 (91.0%)	470 (89.0%)

Values are *n* (%) or median (IQR). Percentages are calculated among those with non-missing data. ^A^ Missing *n* = 2 DD, 3 TD; ^B^ Missing *n* = 4 ASD, 5 TD; ^C^ Missing *n* = 13 ASD, 3 DD, 10 TD; ^D^ Missing *n* = 24 ASD, 9 DD, 14 TD; ^E^ Missing *n* = 9 ASD, 5 DD, 4 TD; ^F^ Missing *n* = 19 ASD, 3 DD, 10 TD; ^G^ Missing *n* = 3 ASD, 3 TD; ^H^ Missing *n* = 3 ASD, 2 DD, 2 TD; ^I^ Missing *n* = 232 ASD, 30 DD, 78 TD; ^J^ Missing *n* = 6 ASD, 1 TD; ^K^ Missing *n* = 12 ASD, 2 DD, 8 TD; ^L^ Missing *n* = 6 ASD, 3 DD, 4 TD; ^M^ Missing *n* = 4 ASD, 3 DD, 2 TD; ^N^ Missing *n* = 56 ASD, 17 DD, 27 TD; ^O^ Missing *n* = 46 ASD, 12 DD, 17 TD; ^P^ Missing *n* = 43 ASD, 12 DD, 16 TD; ^Q^ Missing *n* = 45 ASD, 13 DD, 17 TD; ^R^ Missing *n* = 10 ASD, 4 DD, 3 TD. Abbreviations: ASD, autism spectrum disorder; DD, developmental delay; TD, typically developing.

## Data Availability

The datasets presented in this article are not readily available because the data are part of an ongoing study.

## Supplementary Materials

The following supporting information can be downloaded at: https://www.mdpi.com/article/10.3390/ijerph22071149/s1, Table S1: Distributions (Mean ± SD) of developmental quotient scores of Mullen Scales of Early Learning (MSEL) and Vineland Adaptive Behavior Scales (VABS) for the composite score and each subscale; Table S2: Number (%) of subjects exposed to each application of insecticide by period and case status; Table S3: Beta (95% CI) from linear regression of each insecticide with MSEL Composite DQ; Table S4: Beta (95% CI) from linear regression of each insecticide with VABS Composite DQ; Table S5: Beta (95% CI) and P-values for effect modification of sex on the association between insecticides and MSEL, by diagnosis; Table S6: Beta (95% CI) and P-values for effect modification of sex on the association between insecticides and VABS, by diagnosis; Table S7: Beta (95% CI) for association of Flea/Tick insecticide with MSEL Subscale DQ; Table S8: Beta (95% CI) for association of Flea/Tick insecticide with VABS Subscale DQ; Table S9: Beta (95% CI) for associations of frequency of each insecticide with MSEL Composite DQ; Table S10: Beta (95% CI) for associations of frequency of each insecticide with VABS Composite DQ; Figure S1: Directed acyclic graph (DAG) for the association of prenatal exposure to household insecticides and autism spectrum disorder (ASD), in the CHARGE study population.

## Abbreviations

The following abbreviations are used in this manuscript:

ADI-R	Autism Diagnostic Interview–Revised
ADOS-2	Autism Diagnostic Observation Schedules-2
ASD	Autism spectrum disorder
CHARGE	Childhood Autism Risk from Genetics and the Environment
CI	Confidence interval
DAG	Directed Acyclic Graph
DD	Developmental delay without autism spectrum disorder
DDS	Department of Developmental Services
DQ	Developmental quotients
EPA	Environmental Protection Agency
FDR	False discovery rate
GABA	Gamma-aminobutyric acid
GP	General population
MSEL	Mullen Scales of Early Learning
nAChRs	Nicotinic acetylcholine receptors
NHW	Non-Hispanic white
OP	Organophosphorus
QFDR	Q-value after FDR correction
RC	Regional Centers
SCQ	Social Communication Questionnaire
SES	Socioeconomic status
TD	Typical development
VABS	Vineland Adaptive Behavior Scales
