# Supplementary material for: Pet Flea and Tick Control Exposure During Pregnancy and Early Life Associated with Decreased Cognitive and Adaptive Behaviors in Children with Developmental Delay and Autism Spectrum Disorder
Source: Int J Environ Res Public Health. 2025 Jul 19;22(7):1149. doi: 10.3390/ijerph22071149 (PMC12294743; doi:10.3390/ijerph22071149)
Supplement: Supplementary file 1 [file ijerph-22-01149-s001.zip › ijerph-3697018-supplementary.pdf]

Supplemental Table S1. Distributions (Mean ± SD) of developmental quotient scores of Mullen Scales of Early Learning (MSEL) and Vineland Adaptive Behavior Scales (VABS) for the composite score and each subscale

	ASD (N=810)	DD (N=192)	TD (N=531)
MSEL - Composite Score	61.6 ± 22.8	58.9 ± 19.5	106.2 ± 13.6
MSEL - Receptive Language	54.7 ± 27.2	55.8 ± 19.7	104.3 ± 17.9
MSEL - Expressive Language	54.2 ± 25.4	52.5 ± 23.3	104.2 ± 18.8
MSEL - Visual Receptivity	69.5 ± 25.9	66.2 ± 24.0	112.4 ± 18.1
MSEL - Fine Motor Skills	67.9 ± 21.0	61.2 ± 19.6	103.9 ± 14.8
VABS - Composite Score	55.8 ± 16.0	55.7 ± 18.0	103.9 ± 16.8
VABS - Communication	50.1 ± 23.0	50.8 ± 20.0	106.5 ± 21.8
VABS - Daily Living Skills	53.7 ± 14.6	55.0 ± 19.3	96.8 ± 20.3
VABS - Socialization	45.4 ± 19.0	55.4 ± 22.1	106.5 ± 24.4
VABS - Motor Skills	73.0 ± 19.7	60.4 ± 21.4	106.8 ± 19.7

Abbreviations: ASD, autism spectrum disorder; MSEL, Mullen Scales of Early Learning; VABS, Vineland Adaptive Behavior Scales.

Supplemental Table S2. Number (%) of subjects exposed to each application of insecticide by period and case status.

Application	Period of exposure	Missing N (ASD/DD/TD)	ASD (N=810)	DD (N=192)	TD (N=531)	Total (N=1,533)
Indoor Professionally Applied Insecticide	Index period	15/3/8	116 (14.6%)	19 (10.1%)	43 (8.2%)	178 (11.8%)
	Pre-pregnancy	16/3/10	36 (4.5%)	6 (3.2%)	13 (2.5%)	55 (3.7%)
	Trimester 1	22/3/11	36 (4.6%)	6 (3.2%)	12 (2.3%)	54 (3.6%)
	Trimester 2	23/3/10	45 (5.7%)	5 (2.6%)	16 (3.1%)	66 (4.4%)
	Trimester 3	28/11/12	43 (5.5%)	2 (1.1%)	16 (3.1%)	61 (4.1%)
	Pregnancy	27/3/11	57 (7.3%)	6 (3.2%)	20 (3.8%)	83 (5.6%)
	Year 1	27/3/12	67 (8.6%)	6 (3.2%)	26 (5.0%)	99 (6.6%)
	Year 2	27/3/17	71 (9.1%)	12 (6.3%)	27 (5.3%)	110 (7.4%)
Indoor Non-professionally Applied Insecticide	Index period	7/2/7	310 (38.6%)	76 (40.0%)	198 (37.8%)	584 (38.5%)
	Pre-pregnancy	17/6/9	106 (13.4%)	29 (15.6%)	64 (12.3%)	199 (13.3%)
	Trimester 1	17/6/9	118 (14.9%)	35 (18.8%)	66 (12.6%)	219 (14.6%)
	Trimester 2	18/6/9	129 (16.3%)	35 (18.8%)	79 (15.1%)	243 (16.2%)
	Trimester 3	23/12/11	105 (13.3%)	30 (16.7%)	61 (11.7%)	196 (13.2%)
	Pregnancy	20/6/10	178 (22.5%)	54 (29.0%)	113 (21.7%)	345 (23.0%)
	Year 1	7/2/8	222 (27.6%)	49 (25.8%)	138 (26.4%)	409 (27.0%)
	Year 2	10/2/9	231 (28.9%)	52 (27.4%)	145 (27.8%)	428 (28.3%)
Any Indoor Insecticide	Index period	3/2/5	370 (45.8%)	86 (45.3%)	217 (41.3%)	673 (44.2%)
	Pre-pregnancy	10/6/7	136 (17.0%)	33 (17.7%)	74 (14.1%)	243 (16.1%)
	Trimester 1	11/5/7	144 (18.0%)	40 (21.4%)	76 (14.5%)	260 (17.2%)
	Trimester 2	11/6/7	163 (20.4%)	39 (21.0%)	89 (17.0%)	291 (19.3%)
	Trimester 3	17/12/9	137 (17.3%)	32 (17.8%)	73 (14.0%)	242 (16.2%)
	Pregnancy	11/3/7	217 (27.2%)	57 (30.2%)	123 (23.5%)	397 (26.3%)
	Year 1	3/2/6	265 (32.8%)	54 (28.4%)	152 (29.0%)	471 (30.9%)
	Year 2	5/2/7	274 (34.0%)	61 (32.1%)	163 (31.1%)	498 (32.8%)
Outdoor Non-professional Applied Insecticide	Index period	10/0/7	226 (28.2%)	43 (22.4%)	161 (30.7%)	430 (28.4%)
	Pre-pregnancy	20/2/13	78 (9.9%)	13 (6.8%)	49 (9.5%)	140 (9.3%)
	Trimester 1	20/2/13	87 (11.0%)	19 (10.0%)	53 (10.2%)	159 (10.6%)
	Trimester 2	21/2/12	95 (12.0%)	20 (10.5%)	61 (11.8%)	176 (11.7%)
	Trimester 3	27/10/13	82 (10.5%)	21 (11.5%)	51 (9.8%)	154 (10.4%)
	Pregnancy	23/6/11	133 (16.9%)	31 (16.7%)	100 (19.2%)	264 (17.7%)
	Year 1	12/0/7	176 (22.1%)	33 (17.2%)	117 (22.3%)	326 (21.5%)
	Year 2	12/0/9	186 (23.3%)	34 (17.7%)	126 (24.1%)	346 (22.9%)
Any Outdoor Insecticide	Index period	9/0/7	317 (39.6%)	63 (32.8%)	227 (43.3%)	607 (40.0%)
	Pre-pregnancy	19/2/13	134 (16.9%)	24 (12.6%)	89 (17.2%)	247 (16.5%)
	Trimester 1	19/2/13	144 (18.2%)	30 (15.8%)	92 (17.8%)	266 (17.7%)
	Trimester 2	20/2/12	156 (19.7%)	31 (16.3%)	103 (19.8%)	290 (19.3%)
	Trimester 3	26/10/13	141 (18.0%)	31 (17.0%)	92 (17.8%)	264 (17.8%)
	Pregnancy	22/6/11	194 (24.6%)	42 (22.6%)	145 (27.9%)	381 (25.5%)
	Year 1	11/0/7	252 (31.5%)	47 (24.5%)	166 (31.7%)	465 (30.7%)
	Year 2	11/0/9	265 (33.2%)	51 (26.6%)	177 (33.9%)	493 (32.6%)
Flea/Tick Collar Use	Index period	15/0/3	57 (7.2%)	9 (4.7%)	30 (5.7%)	96 (6.3%)
	Pre-pregnancy	18/3/6	30 (3.8%)	6 (3.2%)	14 (2.7%)	50 (3.3%)
	Trimester 1	18/3/6	29 (3.7%)	6 (3.2%)	16 (3.0%)	51 (3.4%)
	Trimester 2	17/3/8	32 (4.0%)	6 (3.2%)	17 (3.3%)	55 (3.7%)
	Trimester 3	25/11/9	28 (3.6%)	6 (3.3%)	13 (2.5%)	47 (3.2%)
	Pregnancy	22/5/8	37 (4.7%)	7 (3.7%)	22 (4.2%)	66 (4.4%)
	Year 1	19/0/8	33 (4.2%)	8 (4.2%)	17 (3.3%)	58 (3.9%)
	Year 2	19/0/8	36 (4.6%)	6 (3.1%)	15 (2.9%)	57 (3.8%)
Flea/Tick Skin application	Index period	23/2/9	188 (23.9%)	32 (16.8%)	146 (28.0%)	366 (24.4%)
	Pre-pregnancy	41/7/21	111 (14.4%)	19 (10.3%)	77 (15.1%)	207 (14.1%)
	Trimester 1	38/7/22	124 (16.1%)	18 (9.7%)	81 (15.9%)	223 (15.2%)
	Trimester 2	39/8/21	124 (16.1%)	17 (9.2%)	76 (14.9%)	217 (14.8%)
	Trimester 3	46/17/22	116 (15.2%)	14 (8.0%)	79 (15.5%)	209 (14.4%)
	Pregnancy	40/11/21	143 (18.6%)	19 (10.5%)	97 (19.0%)	259 (17.7%)
	Year 1	30/4/12	150 (19.2%)	27 (14.4%)	117 (22.5%)	294 (19.8%)
	Year 2	28/2/13	141 (18.0%)	26 (13.7%)	120 (23.2%)	287 (19.3%)

Flea/Tick Soap, Shampoo, or Powder	Index period	21/2/11	128 (16.2%)	29 (15.3%)	66 (12.7%)	223 (14.9%)
	Pre-pregnancy	31/4/23	76 (9.8%)	21 (11.2%)	32 (6.3%)	129 (8.7%)
	Trimester 1	32/4/24	73 (9.4%)	19 (10.1%)	30 (5.9%)	122 (8.3%)
	Trimester 2	30/4/22	77 (9.9%)	16 (8.5%)	29 (5.7%)	122 (8.3%)
	Trimester 3	40/13/25	73 (9.5%)	14 (7.8%)	30 (5.9%)	117 (8.0%)
	Pregnancy	37/6/23	90 (11.6%)	20 (10.8%)	39 (7.7%)	149 (10.2%)
	Year 1	27/3/14	96 (12.3%)	17 (9.0%)	52 (10.1%)	165 (11.1%)
	Year 2	28/2/15	98 (12.5%)	18 (9.5%)	46 (8.9%)	162 (10.9%)
Any Flea/Tick Control Product	Index period	4/0/1	272 (33.7%)	47 (24.5%)	184 (34.7%)	503 (32.9%)
	Pre-pregnancy	7/1/2	167 (20.8%)	30 (15.7%)	101 (19.1%)	298 (19.6%)
	Trimester 1	7/1/2	179 (22.3%)	32 (16.8%)	107 (20.2%)	318 (20.9%)
	Trimester 2	6/1/3	181 (22.5%)	30 (15.7%)	100 (18.9%)	311 (20.4%)
	Trimester 3	12/9/4	172 (21.6%)	26 (14.2%)	102 (19.4%)	300 (19.9%)
	Pregnancy	8/1/3	206 (25.7%)	34 (17.8%)	128 (24.2%)	368 (24.2%)
	Year 1	6/0/2	209 (26.0%)	37 (19.3%)	147 (27.8%)	393 (25.8%)
	Year 2	6/0/3	211 (26.2%)	34 (17.7%)	148 (28.0%)	393 (25.8%)

Values are N (%). Abbreviations: ASD, autism spectrum disorder; DD, developmental delay; preg, pregnancy; TD, typically developing.

Supplemental Table S3. Beta (95% CI) from linear regression of each insecticide with MSEL Composite DQ

Application	Exposure Period	ASD	DD	TD
Indoor Professionally Applied Insecticide	Pre-pregnancy	7.46 (-0.15, 15.07)	-	-6.08 (-13.54, 1.37)
	Trimester 1	7.23 (-0.39, 14.85)	-	-4.70 (-12.46, 3.05)
	Trimester 2	4.57 (-2.17, 11.31)	-	-2.93 (-9.41, 3.55)
	Trimester 3	4.88 (-2.02, 11.77)	-	-1.91 (-8.41, 4.58)
	Pregnancy	**8.04 (1.93, 14.14)**	-	-2.76 (-8.74, 3.23)
	Year 1	3.52 (-2.17, 9.21)	-	0.60 (-4.54, 5.74)
	Year 2	1.94 (-3.64, 7.51)	-	2.90 (-2.12, 7.92)
Indoor Non-professionally Applied Insecticide	Pre-pregnancy	0.41 (-4.26, 5.08)	-6.65 (-14.85, 1.54)	-0.53 (-3.95, 2.90)
	Trimester 1	2.63 (-1.83, 7.10)	-6.86 (-14.45, 0.73)	-1.93 (-5.37, 1.51)
	Trimester 2	1.63 (-2.62, 5.88)	-2.91 (-10.30, 4.47)	0.15 (-3.05, 3.35)
	Trimester 3	3.27 (-1.37, 7.90)	-7.42 (-15.17, 0.34)	-1.45 (-5.00, 2.10)
	Pregnancy	2.16 (-1.64, 5.97)	-3.54 (-9.87, 2.79)	-1.07 (-3.86, 1.73)
	Year 1	2.61 (-0.93, 6.16)	-0.17 (-6.66, 6.33)	-0.81 (-3.39, 1.76)
	Year 2	1.16 (-2.34, 4.67)	-1.84 (-8.23, 4.54)	-0.55 (-3.10, 2.00)
Any Indoor Insecticide	Pre-pregnancy	1.86 (-2.36, 6.07)	-6.16 (-13.82, 1.49)	-1.06 (-4.30, 2.17)
	Trimester 1	3.56 (-0.56, 7.68)	-6.34 (-13.42, 0.73)	-2.30 (-5.54, 0.95)
	Trimester 2	2.71 (-1.17, 6.59)	-2.98 (-10.07, 4.10)	-0.31 (-3.35, 2.73)
	Trimester 3	4.03 (-0.11, 8.17)	-6.74 (-14.27, 0.79)	-2.31 (-5.57, 0.96)
	Pregnancy	**4.15 (0.60, 7.70)**	-3.42 (-9.61, 2.77)	-1.50 (-4.20, 1.21)
	Year 1	1.89 (-1.48, 5.26)	1.86 (-4.40, 8.12)	-0.95 (-3.44, 1.55)
	Year 2	1.22 (-2.13, 4.57)	-0.06 (-6.10, 5.98)	-0.58 (-3.04, 1.88)
Outdoor Non-professionally Applied Insecticide	Pre-pregnancy	-1.84 (-7.10, 3.42)	-1.20 (-12.75, 10.34)	-0.23 (-4.17, 3.71)
	Trimester 1	2.07 (-2.95, 7.09)	-1.25 (-11.18, 8.69)	-0.87 (-4.73, 2.99)
	Trimester 2	-0.65 (-5.43, 4.13)	-1.37 (-10.98, 8.24)	0.72 (-2.86, 4.29)
	Trimester 3	0.86 (-4.27, 5.98)	-4.34 (-13.68, 5.01)	-2.09 (-5.99, 1.80)
	Pregnancy	-1.34 (-5.54, 2.87)	-0.36 (-8.39, 7.67)	-1.07 (-4.02, 1.88)
	Year 1	1.26 (-2.52, 5.03)	-3.44 (-11.42, 4.53)	0.36 (-2.42, 3.15)
	Year 2	0.30 (-3.42, 4.02)	-1.32 (-9.17, 6.54)	-0.36 (-3.06, 2.34)
Any Outdoor Insecticide	Pre-pregnancy	0.85 (-3.41, 5.11)	-1.39 (-10.14, 7.37)	0.53 (-2.53, 3.59)
	Trimester 1	2.39 (-1.76, 6.55)	-0.48 (-8.54, 7.57)	0.83 (-2.20, 3.85)
	Trimester 2	0.51 (-3.49, 4.52)	-0.61 (-8.56, 7.34)	1.46 (-1.40, 4.31)
	Trimester 3	0.95 (-3.20, 5.11)	-3.74 (-11.67, 4.19)	-0.01 (-2.99, 2.98)
	Pregnancy	-0.56 (-4.30, 3.19)	-0.49 (-7.57, 6.59)	0.17 (-2.39, 2.73)
	Year 1	1.46 (-1.98, 4.90)	-0.19 (-7.03, 6.64)	1.46 (-1.02, 3.94)
	Year 2	0.77 (-2.67, 4.22)	0.32 (-6.45, 7.09)	0.74 (-1.69, 3.17)
Flea/Tick Collar Use	Pre-pregnancy	-5.27 (-13.40, 2.86)	**-19.29 (-36.64, -1.94)**	-1.85 (-8.78, 5.08)
	Trimester 1	-5.82 (-14.09, 2.46)	**-19.29 (-36.64, -1.94)**	-2.63 (-9.09, 3.84)
	Trimester 2	**-7.90 (-15.78, -0.02)**	-14.79 (-32.38, 2.81)	-1.82 (-8.09, 4.45)
	Trimester 3	-5.12 (-13.54, 3.29)	-14.82 (-32.48, 2.85)	-1.29 (-8.46, 5.87)
	Pregnancy	-6.31 (-13.68, 1.06)	-13.52 (-29.59, 2.55)	-3.44 (-9.01, 2.12)
	Year 1	-5.75 (-13.52, 2.02)	-11.49 (-26.55, 3.57)	-0.14 (-6.45, 6.17)
	Year 2	**-8.68 (-16.13, -1.22)**	-15.57 (-31.60, 0.46)	-3.77 (-10.44, 2.90)
Flea/Tick Skin application	Pre-pregnancy	-1.07 (-5.65, 3.51)	**-9.93 (-19.52, -0.33)**	2.27 (-1.03, 5.56)
	Trimester 1	-0.69 (-5.11, 3.73)	**-11.13 (-20.90, -1.36)**	1.81 (-1.45, 5.07)
	Trimester 2	1.33 (-3.05, 5.71)	**-12.86 (-23.01, -2.70)**	1.23 (-2.09, 4.56)
	Trimester 3	-0.23 (-4.73, 4.26)	-10.61 (-21.98, 0.77)	2.71 (-0.54, 5.96)
	Pregnancy	-0.46 (-4.63, 3.71)	**-11.75 (-21.31, -2.20)**	1.98 (-1.04, 4.99)
	Year 1	-0.75 (-4.84, 3.34)	-6.79 (-15.12, 1.53)	2.39 (-0.39, 5.17)
	Year 2	0.83 (-3.33, 4.99)	-4.66 (-12.99, 3.66)	2.13 (-0.61, 4.87)
Flea/Tick Soap, Shampoo, or Powder	Pre-pregnancy	-5.07 (-10.50, 0.37)	-5.76 (-14.95, 3.42)	-2.13 (-6.79, 2.53)
	Trimester 1	-4.86 (-10.37, 0.64)	-0.52 (-10.25, 9.22)	-2.74 (-7.54, 2.07)
	Trimester 2	**-6.45 (-11.81, -1.08)**	-0.72 (-11.39, 9.95)	-3.44 (-8.33, 1.46)
	Trimester 3	**-6.65 (-12.16, -1.13)**	-0.60 (-11.58, 10.39)	-3.06 (-7.88, 1.76)
	Pregnancy	-4.25 (-9.30, 0.79)	0.38 (-9.18, 9.94)	-2.61 (-6.89, 1.67)
	Year 1	**-6.47 (-11.26, -1.67)**	-1.49 (-11.75, 8.78)	-0.78 (-4.55, 2.99)
	Year 2	**-8.87 (-13.62, -4.12)**	-3.14 (-13.11, 6.84)	-1.48 (-5.46, 2.49)
Any Flea/Tick Control Product	Pre-pregnancy	-3.26 (-7.13, 0.62)	-6.40 (-14.31, 1.51)	0.66 (-2.23, 3.55)
	Trimester 1	-2.06 (-5.86, 1.74)	-5.97 (-13.63, 1.70)	0.26 (-2.58, 3.11)
	Trimester 2	-1.94 (-5.70, 1.82)	-6.12 (-14.03, 1.80)	-0.15 (-3.06, 2.77)
	Trimester 3	-2.86 (-6.69, 0.97)	-5.32 (-13.71, 3.06)	1.35 (-1.54, 4.23)
	Pregnancy	-2.03 (-5.66, 1.60)	-4.53 (-12.01, 2.96)	0.03 (-2.63, 2.70)
	Year 1	-2.66 (-6.24, 0.92)	-5.31 (-12.63, 2.01)	1.29 (-1.28, 3.86)
	Year 2	-2.30 (-5.85, 1.26)	-4.28 (-11.70, 3.15)	1.11 (-1.42, 3.65)

Bold typeface indicates statistical significance at *P*< 0.05. Associations for indoor professionally applied insecticides and Flea/Tick collars among DD were not assessed due to small sample sizes. Abbreviations: ASD, autism spectrum disorder; DD, developmental delay; DQ, developmental quotient; MSEL, Mullen Scales of Early Learning; TD, typically developing

Supplemental Table S4. Beta (95% CI) from linear regression of each insecticide with VABS Composite DQ**.**

Application	Exposure Period	ASD	DD	TD
Indoor Professionally Applied Insecticide	Pre-pregnancy	**5.53 (0.23, 10.82)**	-	-3.52 (-12.91, 5.86)
	Trimester 1	5.22 (-0.15, 10.60)	-	-5.85 (-15.60, 3.91)
	Trimester 2	2.76 (-1.96, 7.48)	-	-1.22 (-9.37, 6.92)
	Trimester 3	**5.11 (0.25, 9.96)**	-	-0.29 (-8.46, 7.89)
	Pregnancy	**5.26 (0.98, 9.54)**	-	-0.47 (-8.00, 7.06)
	Year 1	0.78 (-3.21, 4.77)	-	1.84 (-4.62, 8.31)
	Year 2	1.08 (-2.80, 4.95)	-	2.34 (-4.02, 8.69)
Indoor Non-professionally Applied Insecticide	Pre-pregnancy	0.59 (-2.70, 3.87)	-3.03 (-10.65, 4.58)	3.00 (-1.30, 7.29)
	Trimester 1	1.20 (-1.95, 4.36)	1.35 (-5.73, 8.42)	0.00 (-4.33, 4.34)
	Trimester 2	0.07 (-2.93, 3.07)	1.71 (-5.12, 8.54)	2.60 (-1.42, 6.61)
	Trimester 3	-0.56 (-3.84, 2.71)	-2.60 (-9.80, 4.61)	2.79 (-1.68, 7.26)
	Pregnancy	0.22 (-2.47, 2.91)	0.11 (-5.78, 6.00)	1.68 (-1.84, 5.20)
	Year 1	-0.41 (-2.90, 2.08)	0.05 (-5.93, 6.04)	-0.10 (-3.35, 3.16)
	Year 2	0.29 (-2.18, 2.76)	-1.07 (-6.95, 4.82)	-0.53 (-3.74, 2.68)
Any Indoor Insecticide	Pre-pregnancy	2.00 (-0.95, 4.95)	-2.16 (-9.27, 4.96)	1.94 (-2.14, 6.02)
	Trimester 1	2.49 (-0.41, 5.40)	1.48 (-5.12, 8.08)	-0.82 (-4.92, 3.28)
	Trimester 2	1.29 (-1.44, 4.02)	1.85 (-4.70, 8.40)	1.98 (-1.85, 5.80)
	Trimester 3	1.60 (-1.32, 4.53)	-1.78 (-8.77, 5.21)	1.80 (-2.32, 5.92)
	Pregnancy	2.10 (-0.40, 4.61)	0.61 (-5.12, 6.34)	1.37 (-2.04, 4.77)
	Year 1	-0.23 (-2.60, 2.14)	0.85 (-4.93, 6.63)	-0.56 (-3.71, 2.60)
	Year 2	0.60 (-1.75, 2.95)	0.80 (-4.77, 6.36)	-0.62 (-3.72, 2.48)
Outdoor Non-professionally Applied Insecticide	Pre-pregnancy	-0.86 (-4.63, 2.91)	2.14 (-8.46, 12.74)	**5.13 (0.15, 10.10)**
	Trimester 1	0.40 (-3.19, 3.99)	3.62 (-5.48, 12.72)	0.57 (-4.32, 5.47)
	Trimester 2	-0.75 (-4.17, 2.67)	5.47 (-3.28, 14.23)	3.51 (-1.02, 8.03)
	Trimester 3	-0.28 (-3.95, 3.38)	-1.53 (-10.10, 7.05)	1.64 (-3.30, 6.58)
	Pregnancy	-2.40 (-5.39, 0.59)	1.77 (-5.63, 9.18)	1.84 (-1.90, 5.57)
	Year 1	-0.88 (-3.56, 1.80)	0.93 (-6.40, 8.27)	2.70 (-0.79, 6.20)
	Year 2	-0.76 (-3.39, 1.88)	2.41 (-4.77, 9.60)	1.21 (-2.18, 4.61)
Any Outdoor Insecticide	Pre-pregnancy	1.70 (-1.33, 4.74)	2.87 (-5.16, 10.90)	1.68 (-2.19, 5.56)
	Trimester 1	1.61 (-1.35, 4.56)	3.28 (-4.11, 10.67)	0.63 (-3.20, 4.47)
	Trimester 2	0.73 (-2.12, 3.57)	4.62 (-2.64, 11.88)	2.71 (-0.90, 6.33)
	Trimester 3	1.38 (-1.57, 4.33)	-0.01 (-7.30, 7.28)	1.86 (-1.92, 5.64)
	Pregnancy	-0.59 (-3.25, 2.07)	2.33 (-4.19, 8.85)	2.16 (-1.07, 5.39)
	Year 1	0.18 (-2.26, 2.62)	1.77 (-4.50, 8.04)	2.18 (-0.94, 5.29)
	Year 2	0.49 (-1.95, 2.93)	4.06 (-2.12, 10.24)	0.73 (-2.33, 3.79)
Flea/Tick Collar Use	Pre-pregnancy	-4.48 (-10.18, 1.22)	-14.38 (-30.34, 1.59)	2.97 (-5.76, 11.70)
	Trimester 1	-4.01 (-9.81, 1.80)	-14.38 (-30.34, 1.59)	1.70 (-6.47, 9.86)
	Trimester 2	-4.30 (-9.82, 1.23)	-8.88 (-25.06, 7.31)	4.24 (-3.64, 12.13)
	Trimester 3	-4.60 (-10.50, 1.29)	-8.90 (-25.15, 7.35)	0.63 (-8.43, 9.68)
	Pregnancy	-4.45 (-9.63, 0.72)	-9.75 (-24.61, 5.10)	3.44 (-3.58, 10.47)
	Year 1	-2.50 (-7.95, 2.94)	-7.00 (-20.87, 6.87)	1.97 (-5.97, 9.91)
	Year 2	-2.25 (-7.49, 2.98)	-10.38 (-25.17, 4.41)	-1.61 (-10.01, 6.80)
Flea/Tick Skin application	Pre-pregnancy	1.03 (-2.18, 4.24)	**-10.61 (-19.26, -1.96)**	3.08 (-1.03, 7.19)
	Trimester 1	1.46 (-1.64, 4.55)	**-10.41 (-19.26, -1.57)**	0.84 (-3.24, 4.92)
	Trimester 2	1.85 (-1.22, 4.92)	**-11.73 (-20.92, -2.54)**	0.67 (-3.48, 4.82)
	Trimester 3	2.02 (-1.13, 5.17)	**-10.97 (-21.24, -0.71)**	1.24 (-2.85, 5.33)
	Pregnancy	1.11 (-1.81, 4.02)	**-11.09 (-19.80, -2.39)**	0.89 (-2.89, 4.67)
	Year 1	1.07 (-1.79, 3.94)	-3.77 (-11.39, 3.84)	-0.77 (-4.25, 2.72)
	Year 2	1.30 (-1.61, 4.21)	-2.77 (-10.36, 4.83)	-0.32 (-3.76, 3.12)
Flea/Tick Soap, Shampoo, or Powder	Pre-pregnancy	-2.73 (-6.51, 1.06)	**-9.08 (-17.41, -0.76)**	0.12 (-5.80, 6.04)
	Trimester 1	-2.18 (-6.01, 1.65)	-6.33 (-15.18, 2.52)	-1.18 (-7.26, 4.91)
	Trimester 2	-3.25 (-6.99, 0.49)	-6.93 (-16.63, 2.76)	-1.58 (-7.77, 4.60)
	Trimester 3	-3.08 (-6.93, 0.77)	-6.85 (-16.84, 3.14)	-0.74 (-6.85, 5.37)
	Pregnancy	-2.10 (-5.63, 1.42)	-6.38 (-15.06, 2.31)	0.75 (-4.66, 6.15)
	Year 1	-2.44 (-5.80, 0.92)	-3.74 (-13.18, 5.70)	0.43 (-4.32, 5.18)
	Year 2	**-4.37 (-7.69, -1.04)**	-3.21 (-12.38, 5.95)	-2.04 (-7.05, 2.98)
Any Flea/Tick Control Product	Pre-pregnancy	-1.07 (-3.79, 1.64)	**-9.07 (-16.22, -1.92)**	1.53 (-2.09, 5.16)
	Trimester 1	-0.14 (-2.80, 2.51)	**-8.23 (-15.16, -1.29)**	0.69 (-2.89, 4.27)
	Trimester 2	-0.33 (-2.96, 2.30)	**-7.69 (-14.88, -0.51)**	0.47 (-3.18, 4.12)
	Trimester 3	-0.75 (-3.43, 1.93)	**-7.65 (-15.25, -0.05)**	0.59 (-3.04, 4.22)
	Pregnancy	-0.62 (-3.16, 1.92)	**-7.40 (-14.17, -0.63)**	0.92 (-2.43, 4.27)
	Year 1	-0.39 (-2.90, 2.11)	-3.75 (-10.49, 2.98)	-1.12 (-4.35, 2.11)
	Year 2	-0.54 (-3.02, 1.94)	-2.71 (-9.54, 4.12)	-0.86 (-4.04, 2.33)

Bold typeface indicates statistical significance at $P < 0.05$. Associations for indoor professionally applied insecticides and Flea/Tick collars among DD could not be assessed due to small sample sizes. Abbreviations: ASD, autism spectrum disorder; DD, developmental delay; DQ, developmental quotient; TD, typically developing; VABS, Vineland Adaptive Behavior Scales.

Supplemental Table S5. Beta (95% CI) and P-values for effect modification of sex on the association between insecticides and MSEL, by diagnosis.

		ASD			DD			TD		
Application	Exposure Period	Males (*n* = 653)	Females (*n* = 130)	P_{int}	Males (*n* = 129)	Females (*n* = 56)	P_{int}	Males (*n* = 422)	Females (*n* = 98)	P_{int}
Indoor Professionally Applied Insecticide	Pre-pregnancy	5.32 (-2.78, 13.41)	-	-	7.50 (-15.08, 30.08)	-	-	-7.91 (-15.65, -0.17)	-	-
	Trimester 1	7.24 (-0.91, 15.39)	-	-	-1.16 (-24.45, 22.14)	-	-	-6.60 (-14.69, 1.48)	-	-
	Trimester 2	2.17 (-5.60, 9.94)	-	-	-1.18 (-29.01, 26.66)	-	-	-3.88 (-10.54, 2.79)	-	-
	Trimester 3	5.02 (-2.46, 12.51)	-	-	-1.03 (-28.82, 26.76)	-	-	-2.83 (-9.52, 3.85)	-	-
	Pregnancy	**7.20 (0.36, 14.05)**	-	-	-1.16 (-24.45, 22.14)	-	-	-3.42 (-9.55, 2.70)	-	-
	Year 1	3.28 (-2.92, 9.47)	-	-	3.03 (-16.00, 22.05)	-	-	-0.71 (-6.53, 5.10)	-	-
	Year 2	1.67 (-4.43, 7.78)	-	-	5.97 (-7.91, 19.86)	-	-	1.89 (-3.74, 7.53)	-	-
Indoor Non-professionally Applied Insecticide	Pre-pregnancy	1.35 (-3.50, 6.19)	-11.12 (-30.95, 8.71)	0.23	-5.46 (-14.58, 3.66)	-14.74 (-31.05, 1.57)	0.32	-1.91 (-5.63, 1.82)	6.23 (-1.85, 14.32)	**0.07**
	Trimester 1	3.79 (-0.95, 8.52)	-5.63 (-19.31, 8.05)	0.20	-7.97 (-16.71, 0.78)	-5.74 (-20.27, 8.79)	0.80	-3.47 (-7.22, 0.27)	5.31 (-2.81, 13.44)	**0.05**
	Trimester 2	3.11 (-1.41, 7.63)	-8.47 (-20.78, 3.84)	0.08	-1.32 (-10.27, 7.64)	-5.80 (-18.85, 7.26)	0.58	-0.28 (-3.82, 3.27)	1.50 (-5.61, 8.62)	0.66
	Trimester 3	3.32 (-1.59, 8.22)	4.00 (-10.32, 18.32)	0.93	-5.47 (-15.57, 4.63)	-7.31 (-19.74, 5.11)	0.82	0.04 (-3.72, 3.79)	-9.86 (-19.86, 0.14)	**0.07**
	Pregnancy	2.76 (-1.30, 6.82)	-1.06 (-12.12, 9.99)	0.52	-1.33 (-9.21, 6.55)	-6.27 (-17.17, 4.63)	0.48	-1.97 (-5.05, 1.12)	2.28 (-3.99, 8.56)	0.23
	Year 1	3.61 (-0.19, 7.41)	-2.96 (-13.10, 7.18)	0.23	-0.58 (-8.26, 7.09)	-0.22 (-12.13, 11.68)	0.96	-2.00 (-4.84, 0.85)	3.54 (-2.17, 9.25)	**0.09**
	Year 2	2.11 (-1.69, 5.92)	-3.74 (-12.80, 5.32)	0.24	1.25 (-6.59, 9.08)	-5.90 (-16.78, 4.98)	0.30	-0.82 (-3.61, 1.96)	1.29 (-4.70, 7.27)	0.53
Any Indoor Insecticide	Pre-pregnancy	1.77 (-2.65, 6.20)	5.38 (-9.71, 20.46)	0.65	-4.89 (-13.67, 3.89)	-11.73 (-26.19, 2.74)	0.42	-2.66 (-6.16, 0.84)	7.34 (-0.44, 15.11)	**0.02**
	Trimester 1	4.14 (-0.25, 8.53)	0.42 (-11.88, 12.73)	0.58	-7.20 (-15.45, 1.05)	-5.07 (-18.12, 7.98)	0.79	-4.05 (-7.57, -0.53)	6.49 (-1.31, 14.29)	**0.02**
	Trimester 2	2.81 (-1.38, 7.01)	2.57 (-7.62, 12.77)	0.97	-1.32 (-9.90, 7.26)	-5.41 (-17.60, 6.77)	0.59	-0.65 (-3.98, 2.68)	1.47 (-5.65, 8.59)	0.60
	Trimester 3	4.08 (-0.33, 8.48)	4.83 (-7.46, 17.12)	0.91	-4.94 (-14.46, 4.58)	-7.36 (-19.78, 5.07)	0.76	-1.40 (-4.86, 2.06)	-6.41 (-15.83, 3.01)	0.33
	Pregnancy	**3.93 (0.10, 7.76)**	6.46 (-3.07, 15.98)	0.63	-1.29 (-8.91, 6.33)	-6.13 (-16.81, 4.55)	0.47	-2.30 (-5.25, 0.66)	2.26 (-4.00, 8.51)	0.20
	Year 1	2.91 (-0.73, 6.55)	-3.45 (-12.52, 5.62)	0.20	-0.12 (-7.48, 7.24)	5.08 (-6.45, 16.62)	0.46	-2.13 (-4.89, 0.64)	3.06 (-2.45, 8.58)	0.10
	Year 2	1.97 (-1.68, 5.61)	-2.45 (-10.94, 6.04)	0.35	1.80 (-5.52, 9.12)	-1.91 (-12.34, 8.52)	0.57	-0.89 (-3.57, 1.80)	1.47 (-4.31, 7.25)	0.47
Outdoor Non-professionally Applied Insecticide	Pre-pregnancy	-1.48 (-7.13, 4.18)	-3.67 (-17.98, 10.64)	0.78	-0.41 (-12.93, 12.12)	-	-	-0.98 (-5.35, 3.38)	2.38 (-6.11, 10.87)	0.49
	Trimester 1	3.04 (-2.48, 8.56)	-2.80 (-14.76, 9.17)	0.38	-1.65 (-12.96, 9.66)	-	-	-2.72 (-7.05, 1.61)	5.17 (-2.96, 13.31)	**0.09**
	Trimester 2	0.71 (-4.48, 5.90)	-7.82 (-20.13, 4.50)	0.21	0.55 (-10.71, 11.80)	-	-	0.62 (-3.26, 4.50)	2.58 (-6.39, 11.54)	0.69
	Trimester 3	2.21 (-3.34, 7.76)	-6.67 (-19.86, 6.51)	0.22	-3.75 (-15.00, 7.50)	-	-	-3.05 (-7.27, 1.18)	4.01 (-5.39, 13.42)	0.18
	Pregnancy	-0.60 (-5.15, 3.95)	-5.11 (-15.98, 5.76)	0.45	0.10 (-8.95, 9.15)	-	-	-1.80 (-5.03, 1.44)	2.71 (-4.08, 9.50)	0.24
	Year 1	2.24 (-1.85, 6.33)	-3.83 (-13.43, 5.76)	0.25	-3.83 (-12.83, 5.16)	-	-	0.49 (-2.56, 3.54)	0.73 (-5.79, 7.26)	0.95
	Year 2	1.26 (-2.76, 5.28)	-4.61 (-14.34, 5.13)	0.27	-1.40 (-10.35, 7.55)	-	-	-0.09 (-3.06, 2.87)	-1.35 (-7.49, 4.80)	0.72
Any Outdoor Insecticide	Pre-pregnancy	1.56 (-3.00, 6.11)	-2.95 (-14.61, 8.71)	0.48	-2.30 (-12.43, 7.84)	0.39 (-16.11, 16.89)	0.79	-0.25 (-3.55, 3.05)	5.48 (-1.87, 12.82)	0.16
	Trimester 1	3.84 (-0.66, 8.35)	-5.32 (-15.41, 4.77)	0.10	-1.23 (-10.49, 8.04)	0.33 (-14.99, 15.65)	0.86	-0.49 (-3.76, 2.77)	**8.59 (1.25, 15.93)**	**0.03**
	Trimester 2	2.37 (-1.96, 6.69)	-9.39 (-19.32, 0.53)	**0.03**	0.20 (-9.08, 9.47)	-3.40 (-17.94, 11.14)	0.68	1.15 (-1.90, 4.20)	5.79 (-1.81, 13.38)	0.27
	Trimester 3	2.62 (-1.85, 7.08)	-8.58 (-19.22, 2.06)	**0.06**	-4.66 (-14.23, 4.91)	-1.50 (-14.74, 11.73)	0.70	-0.79 (-3.98, 2.40)	7.15 (-0.70, 15.01)	**0.07**
	Pregnancy	0.51 (-3.52, 4.55)	-6.34 (-15.75, 3.07)	0.18	-0.56 (-8.80, 7.69)	-1.58 (-14.36, 11.20)	0.89	-0.48 (-3.23, 2.28)	5.70 (-0.69, 12.09)	**0.08**
	Year 1	2.45 (-1.27, 6.17)	-3.67 (-12.22, 4.88)	0.19	-4.14 (-12.28, 4.01)	8.70 (-2.83, 20.23)	**0.07**	1.06 (-1.64, 3.76)	4.29 (-1.53, 10.10)	0.32
	Year 2	2.11 (-1.59, 5.82)	-6.24 (-14.95, 2.48)	**0.08**	-2.63 (-10.68, 5.41)	6.67 (-4.75, 18.09)	0.19	0.36 (-2.29, 3.01)	2.47 (-3.12, 8.06)	0.50
Flea/Tick Collar Use	Pre-pregnancy	-2.60 (-11.33, 6.13)	-	-	**-23.31 (-45.25, -1.38)**	-	-	-3.20 (-10.66, 4.26)	-	-
	Trimester 1	-5.19 (-14.14, 3.75)	-	-	**-23.31 (-45.25, -1.38)**	-	-	-3.84 (-10.74, 3.07)	-	-
	Trimester 2	-7.12 (-15.57, 1.33)	-	-	-15.72 (-38.02, 6.59)	-	-	-0.97 (-8.13, 6.19)	-	-
	Trimester 3	-3.87 (-12.98, 5.25)	-	-	-16.59 (-38.94, 5.75)	-	-	-2.84 (-10.61, 4.94)	-	-
	Pregnancy	-6.12 (-14.19, 1.94)	-	-	-15.04 (-34.52, 4.43)	-	-	-3.00 (-9.14, 3.14)	-	-
	Year 1	-3.66 (-11.82, 4.49)	-	-	-9.58 (-29.04, 9.89)	-	-	-2.06 (-8.97, 4.85)	-	-
	Year 2	-6.95 (-15.10, 1.20)	-	-	-15.76 (-37.79, 6.26)	-	-	-5.23 (-12.36, 1.90)	-	-

Flea/Tick Skin application	Pre-pregnancy	1.69 (-3.41, 6.78)	**-12.49 (-22.57, -2.40)**	**0.01**	**-10.32 (-20.41, -0.23)**	-	-	2.30 (-1.37, 5.96)	1.85 (-5.15, 8.86)	0.91
	Trimester 1	1.94 (-2.97, 6.86)	**-11.63 (-21.41, -1.85)**	**0.02**	**-12.90 (-22.92, -2.88)**	-	-	1.07 (-2.57, 4.71)	4.40 (-2.46, 11.26)	0.40
	Trimester 2	3.62 (-1.24, 8.48)	-8.37 (-18.14, 1.41)	**0.03**	**-13.14 (-23.85, -2.43)**	-	-	0.79 (-2.93, 4.51)	2.09 (-4.77, 8.95)	0.74
	Trimester 3	2.48 (-2.57, 7.53)	**-10.75 (-20.40, -1.09)**	**0.02**	**-11.88 (-23.58, -0.17)**	-	-	2.48 (-1.20, 6.16)	2.51 (-4.11, 9.13)	>0.99
	Pregnancy	2.31 (-2.29, 6.91)	**-12.51 (-21.88, -3.13)**	**0.01**	**-12.38 (-22.45, -2.31)**	-	-	1.48 (-1.85, 4.80)	3.86 (-2.65, 10.37)	0.52
	Year 1	1.71 (-2.81, 6.22)	**-11.48 (-20.64, -2.31)**	**0.01**	-7.95 (-17.01, 1.10)	-	-	2.30 (-0.76, 5.35)	2.88 (-3.25, 9.01)	0.87
	Year 2	2.52 (-2.15, 7.18)	-6.16 (-15.25, 2.92)	**0.09**	-5.87 (-15.14, 3.40)	-	-	2.07 (-0.93, 5.07)	3.20 (-3.10, 9.49)	0.75
Flea/Tick Soap, Shampoo, or Powder	Pre-pregnancy	-2.96 (-8.70, 2.78)	**-22.20 (-39.11, -5.29)**	**0.04**	-4.92 (-16.53, 6.69)	-4.64 (-19.34, 10.07)	0.98	-2.59 (-7.92, 2.74)	-1.85 (-11.23, 7.52)	0.89
	Trimester 1	-2.42 (-8.29, 3.44)	**-21.95 (-37.84, -6.07)**	**0.02**	3.56 (-8.46, 15.59)	-4.89 (-20.56, 10.78)	0.40	-1.74 (-6.97, 3.49)	-7.42 (-19.07, 4.23)	0.38
	Trimester 2	-5.38 (-11.27, 0.50)	-11.85 (-25.06, 1.36)	0.38	5.85 (-8.25, 19.96)	-4.84 (-20.50, 10.81)	0.31	-3.40 (-8.86, 2.07)	-4.13 (-14.85, 6.58)	0.90
	Trimester 3	-5.65 (-11.67, 0.38)	-11.95 (-25.68, 1.78)	0.41	3.25 (-11.72, 18.22)	-0.55 (-16.21, 15.12)	0.73	-3.80 (-9.16, 1.56)	-0.55 (-11.28, 10.17)	0.60
	Pregnancy	-2.89 (-8.37, 2.60)	-11.61 (-24.37, 1.14)	0.22	3.45 (-8.65, 15.56)	-1.24 (-16.12, 13.63)	0.63	-2.54 (-7.30, 2.22)	-3.52 (-12.95, 5.92)	0.86
	Year 1	**-5.45 (-10.65, -0.25)**	-12.17 (-24.85, 0.51)	0.34	-1.64 (-13.79, 10.50)	-1.36 (-19.18, 16.47)	0.98	-1.60 (-5.73, 2.52)	3.30 (-5.70, 12.30)	0.33
	Year 2	**-7.29 (-12.46, -2.13)**	**-17.48 (-29.71, -5.24)**	0.13	-4.21 (-16.28, 7.87)	0.16 (-16.15, 16.48)	0.67	-1.28 (-5.55, 2.99)	-1.29 (-12.06, 9.49)	>0.99
Any Flea/Tick Control Product	Pre-pregnancy	0.08 (-2.86, 3.03)	**-7.41 (-14.19, -0.62)**	**0.047**	**-9.06 (-17.39, -0.74)**	-9.27 (-22.38, 3.83)	0.98	0.24 (-3.75, 4.23)	6.46 (-1.48, 14.39)	0.17
	Trimester 1	1.21 (-1.69, 4.11)	**-6.84 (-13.26, -0.43)**	**0.03**	**-8.24 (-16.22, -0.26)**	-9.25 (-22.38, 3.88)	0.90	-0.30 (-4.24, 3.64)	4.70 (-3.11, 12.52)	0.26
	Trimester 2	0.67 (-2.22, 3.56)	-5.04 (-11.32, 1.24)	0.11	-7.16 (-15.52, 1.20)	-9.28 (-22.45, 3.90)	0.79	-1.74 (-5.75, 2.28)	**8.09 (0.32, 15.85)**	**0.03**
	Trimester 3	0.49 (-2.46, 3.45)	-6.10 (-12.32, 0.11)	0.06	-7.98 (-16.94, 0.98)	-6.39 (-19.58, 6.80)	0.84	-1.32 (-5.32, 2.69)	6.75 (-0.99, 14.50)	0.07
	Pregnancy	0.63 (-2.15, 3.40)	**-6.65 (-12.69, -0.61)**	**0.03**	-7.42 (-15.29, 0.45)	-8.15 (-20.69, 4.38)	0.92	-0.53 (-4.19, 3.12)	6.98 (-0.42, 14.38)	0.07
	Year 1	0.78 (-1.95, 3.52)	**-6.18 (-12.22, -0.13)**	**0.04**	-5.21 (-12.96, 2.54)	-2.24 (-14.87, 10.40)	0.69	-2.33 (-5.83, 1.18)	4.47 (-2.72, 11.65)	0.09
	Year 2	-0.07 (-2.81, 2.66)	-2.60 (-8.51, 3.31)	0.45	-3.64 (-11.62, 4.35)	-1.67 (-14.23, 10.90)	0.80	-0.83 (-4.29, 2.63)	0.43 (-6.96, 7.82)	0.76

Bold typeface indicates statistical significance at $P < 0.05$. Dashes indicate that associations could not be estimated due to small sample sizes. Abbreviations: ASD, autism spectrum disorder; MSEL, Mullen Scales of Early Learning; P_{int}, *P*-value for interaction; VABS, Vineland Adaptive Behavior Scales.

Supplemental Table S6. Beta (95% CI) and P-values for effect modification of sex on the association between insecticides and VABS, by diagnosis.

		ASD			DD			TD		
		Males	Females		Males	Females		Males	Females	
Application	Exposure Period	(*n* = 657)	(*n* = 129)	P_{int}	(*n* = 129)	(*n* = 56)	P_{int}	(*n* = 422)	(*n* = 98)	P_{int}
Indoor Professionally Applied Insecticide	Pre-pregnancy	4.62 (-1.01, 10.25)	-	-	5.55 (-15.16, 26.26)	-	-	-4.79 (-14.51, 4.92)	-	-
	Trimester 1	5.22 (-0.53, 10.98)	-	-	4.16 (-17.28, 25.59)	-	-	-7.57 (-17.70, 2.55)	-	-
	Trimester 2	2.13 (-3.30, 7.56)	-	-	5.47 (-20.04, 30.99)	-	-	-1.69 (-10.04, 6.67)	-	-
	Trimester 3	**5.63 (0.36, 10.90)**	-	-	5.41 (-20.04, 30.86)	-	-	-0.68 (-9.06, 7.69)	-	-
	Pregnancy	**5.36 (0.57, 10.15)**	-	-	4.16 (-17.28, 25.59)	-	-	-0.62 (-8.30, 7.06)	-	-
	Year 1	0.54 (-3.81, 4.88)	-	-	7.00 (-10.66, 24.65)	-	-	2.51 (-4.76, 9.79)	-	-
	Year 2	1.16 (-3.07, 5.38)	0.63 (-9.08, 10.34)	0.92	5.45 (-7.27, 18.18)	11.86 (-6.35, 30.07)	0.57	3.41 (-3.69, 10.51)	-2.80 (-16.33, 10.73)	0.42
Indoor Non-professionally Applied Insecticide	Pre-pregnancy	0.93 (-2.47, 4.33)	-7.54 (-21.55, 6.48)	0.25	-2.37 (-10.86, 6.13)	-9.09 (-24.26, 6.09)	0.44	2.69 (-1.97, 7.36)	4.79 (-5.34, 14.91)	0.71
	Trimester 1	1.79 (-1.56, 5.14)	-4.33 (-14.01, 5.35)	0.24	1.49 (-6.67, 9.65)	-0.82 (-14.38, 12.73)	0.77	-1.53 (-6.23, 3.17)	7.20 (-3.00, 17.39)	0.13
	Trimester 2	0.96 (-2.23, 4.15)	-7.17 (-15.87, 1.54)	0.09	1.93 (-6.35, 10.21)	1.67 (-10.39, 13.73)	0.97	2.82 (-1.61, 7.25)	1.14 (-7.76, 10.03)	0.74
	Trimester 3	-0.81 (-4.27, 2.65)	0.82 (-9.31, 10.94)	0.76	-1.67 (-11.00, 7.67)	-0.95 (-12.44, 10.54)	0.93	3.91 (-0.79, 8.62)	-1.02 (-13.53, 11.49)	0.47
	Pregnancy	0.46 (-2.41, 3.33)	-2.31 (-10.14, 5.51)	0.51	1.88 (-5.42, 9.18)	-1.81 (-11.92, 8.29)	0.57	1.24 (-2.63, 5.10)	2.87 (-4.99, 10.73)	0.71
	Year 1	-0.28 (-2.95, 2.40)	-2.26 (-9.43, 4.91)	0.61	-1.06 (-8.11, 5.99)	1.66 (-9.28, 12.60)	0.68	-0.52 (-4.10, 3.07)	0.92 (-6.27, 8.12)	0.72
	Year 2	0.47 (-2.21, 3.15)	-1.00 (-7.40, 5.40)	0.68	0.75 (-6.47, 7.98)	-2.75 (-12.77, 7.28)	0.58	-0.85 (-4.34, 2.64)	1.86 (-5.63, 9.35)	0.52
Any Indoor Insecticide	Pre-pregnancy	1.91 (-1.19, 5.01)	1.51 (-9.13, 12.15)	0.94	-1.46 (-9.64, 6.71)	-6.02 (-19.47, 7.44)	0.57	1.29 (-3.11, 5.70)	5.89 (-3.90, 15.67)	0.40
	Trimester 1	2.70 (-0.40, 5.80)	0.16 (-8.53, 8.85)	0.59	1.90 (-5.79, 9.60)	-0.42 (-12.58, 11.74)	0.75	-2.58 (-7.01, 1.85)	8.12 (-1.69, 17.93)	0.05
	Trimester 2	1.46 (-1.49, 4.41)	-0.01 (-7.21, 7.19)	0.71	2.36 (-5.57, 10.29)	1.71 (-9.55, 12.96)	0.93	2.27 (-1.90, 6.44)	1.05 (-7.87, 9.96)	0.81
	Trimester 3	1.39 (-1.72, 4.50)	2.54 (-6.15, 11.22)	0.81	-0.82 (-9.63, 7.98)	-0.99 (-12.49, 10.50)	0.98	2.54 (-1.80, 6.88)	1.16 (-10.64, 12.97)	0.83
	Pregnancy	1.95 (-0.75, 4.65)	2.59 (-4.15, 9.33)	0.86	2.36 (-4.67, 9.39)	-1.37 (-11.22, 8.47)	0.55	1.09 (-2.62, 4.80)	2.85 (-5.01, 10.70)	0.69
	Year 1	0.11 (-2.44, 2.67)	-3.14 (-9.54, 3.27)	0.35	0.12 (-6.67, 6.91)	1.05 (-9.58, 11.68)	0.89	-0.64 (-4.13, 2.84)	-1.33 (-8.28, 5.62)	0.86
	Year 2	0.97 (-1.59, 3.52)	-1.67 (-7.66, 4.32)	0.43	1.31 (-5.42, 8.04)	1.62 (-7.97, 11.21)	0.96	-0.46 (-3.83, 2.91)	-0.40 (-7.65, 6.85)	0.99
Outdoor Non-professionally Applied Insecticide	Pre-pregnancy	-0.83 (-4.87, 3.20)	-1.23 (-11.89, 9.44)	0.95	3.60 (-7.88, 15.09)	-	-	5.18 (-0.31, 10.68)	4.43 (-6.26, 15.11)	0.90
	Trimester 1	1.14 (-2.80, 5.08)	-3.23 (-11.98, 5.52)	0.37	4.09 (-6.27, 14.44)	-	-	-1.07 (-6.54, 4.40)	5.60 (-4.66, 15.87)	0.26
	Trimester 2	-0.31 (-4.02, 3.39)	-3.47 (-12.50, 5.56)	0.53	6.46 (-3.78, 16.69)	-	-	4.02 (-0.87, 8.91)	2.76 (-8.52, 14.05)	0.84
	Trimester 3	0.38 (-3.58, 4.33)	-4.38 (-14.11, 5.35)	0.37	-1.82 (-12.09, 8.44)	-	-	2.70 (-2.65, 8.04)	-1.52 (-13.40, 10.36)	0.52
	Pregnancy	-1.81 (-5.04, 1.42)	-6.10 (-13.97, 1.78)	0.32	1.52 (-6.80, 9.85)	-	-	1.66 (-2.42, 5.75)	3.43 (-5.15, 12.00)	0.72
	Year 1	-0.03 (-2.93, 2.87)	-5.87 (-12.76, 1.01)	0.12	0.66 (-7.60, 8.93)	-	-	3.04 (-0.77, 6.85)	2.60 (-5.55, 10.75)	0.92
	Year 2	-0.04 (-2.89, 2.81)	-5.38 (-12.38, 1.62)	0.16	3.46 (-4.70, 11.62)	-	-	0.78 (-2.94, 4.49)	3.46 (-4.24, 11.15)	0.54
Any Outdoor Insecticide	Pre-pregnancy	1.82 (-1.41, 5.06)	0.61 (-7.89, 9.11)	0.79	2.08 (-7.21, 11.38)	4.31 (-10.81, 19.42)	0.81	1.00 (-3.17, 5.17)	6.60 (-2.68, 15.89)	0.28
	Trimester 1	2.64 (-0.57, 5.84)	-3.89 (-11.17, 3.39)	0.11	3.32 (-5.18, 11.81)	1.98 (-12.04, 16.00)	0.87	-0.72 (-4.85, 3.40)	9.09 (-0.17, 18.35)	0.06
	Trimester 2	1.65 (-1.44, 4.73)	-4.47 (-11.63, 2.70)	0.12	4.99 (-3.47, 13.45)	2.90 (-10.37, 16.17)	0.79	2.82 (-1.03, 6.68)	5.62 (-3.97, 15.20)	0.60
	Trimester 3	2.41 (-0.76, 5.59)	-5.02 (-12.72, 2.68)	**0.08**	-0.65 (-9.41, 8.11)	1.63 (-10.52, 13.77)	0.76	2.32 (-1.71, 6.36)	2.79 (-7.13, 12.71)	0.93
	Pregnancy	0.22 (-2.65, 3.08)	-5.42 (-12.18, 1.34)	0.13	2.01 (-5.57, 9.59)	1.89 (-9.85, 13.64)	0.99	1.91 (-1.57, 5.38)	6.52 (-1.54, 14.58)	0.30
	Year 1	0.89 (-1.74, 3.53)	-3.88 (-9.98, 2.23)	0.16	1.66 (-5.86, 9.19)	2.88 (-7.77, 13.54)	0.85	1.91 (-1.47, 5.30)	4.84 (-2.43, 12.12)	0.47
	Year 2	1.26 (-1.36, 3.89)	-4.39 (-10.62, 1.84)	**0.10**	3.34 (-4.01, 10.69)	5.81 (-4.64, 16.27)	0.70	-0.32 (-3.64, 3.00)	5.49 (-1.51, 12.48)	0.14
Flea/Tick Collar Use	Pre-pregnancy	-3.64 (-9.77, 2.49)	-	-	-18.43 (-38.58, 1.71)	-	-	0.69 (-8.65, 10.02)	-	-
	Trimester 1	-3.63 (-9.90, 2.65)	-	-	-18.43 (-38.58, 1.71)	-	-	-0.31 (-8.98, 8.35)	-	-
	Trimester 2	-3.61 (-9.54, 2.33)	-	-	-9.20 (-29.70, 11.30)	-	-	0.11 (-8.82, 9.05)	-	-
	Trimester 3	-3.84 (-10.22, 2.55)	-	-	-9.80 (-30.30, 10.71)	-	-	-2.47 (-12.22, 7.27)	-	-
	Pregnancy	-4.01 (-9.68, 1.65)	-	-	-11.62 (-29.59, 6.35)	-	-	0.56 (-7.13, 8.25)	-	-
	Year 1	-1.13 (-6.85, 4.59)	-	-	-6.72 (-24.64, 11.20)	-	-	-1.57 (-10.20, 7.06)	-	-
	Year 2	-0.33 (-6.05, 5.40)	-	-	-12.05 (-32.34, 8.24)	-	-	-4.31 (-13.23, 4.61)	-	-

Flea/Tick Skin application	Pre-pregnancy	2.54 (-1.02, 6.11)	-4.99 (-12.18, 2.19)	**0.06**	-11.09 (-20.18, -1.99)	-	-	2.36 (-2.19, 6.92)	5.34 (-3.36, 14.05)	0.55
	Trimester 1	2.78 (-0.65, 6.21)	-3.87 (-10.82, 3.09)	**0.09**	-11.78 (-20.84, -2.72)	-	-	0.14 (-4.40, 4.67)	3.27 (-5.28, 11.82)	0.52
	Trimester 2	2.82 (-0.58, 6.22)	-2.04 (-8.99, 4.91)	0.22	-11.86 (-21.55, -2.16)	-	-	-1.30 (-5.92, 3.31)	6.63 (-1.89, 15.15)	0.10
	Trimester 3	**3.57 (0.04, 7.09)**	-3.48 (-10.33, 3.37)	**0.07**	-11.98 (-22.52, -1.45)	-	-	-0.54 (-5.14, 4.05)	5.60 (-2.67, 13.87)	0.20
	Pregnancy	2.38 (-0.83, 5.60)	-4.33 (-11.00, 2.33)	**0.07**	-11.66 (-20.82, -2.50)	-	-	-0.31 (-4.46, 3.84)	5.54 (-2.59, 13.66)	0.20
	Year 1	2.30 (-0.87, 5.46)	-4.01 (-10.51, 2.50)	**0.09**	-5.79 (-14.08, 2.51)	-	-	-1.77 (-5.58, 2.04)	3.62 (-4.03, 11.28)	0.21
	Year 2	1.81 (-1.45, 5.06)	-0.34 (-6.78, 6.10)	0.56	-4.42 (-12.88, 4.04)	-	-	-0.37 (-4.12, 3.39)	1.07 (-6.81, 8.94)	0.75
Flea/Tick Soap, Shampoo, or Powder	Pre-pregnancy	-1.88 (-5.88, 2.12)	-10.73 (-22.60, 1.13)	0.17	-7.71 (-18.24, 2.83)	-9.09 (-22.42, 4.25)	0.87	-3.38 (-10.09, 3.33)	8.93 (-2.87, 20.72)	0.08
	Trimester 1	-0.87 (-4.96, 3.21)	**-12.12 (-23.27, -0.97)**	**0.06**	-2.28 (-13.24, 8.67)	-10.94 (-25.20, 3.31)	0.34	-0.02 (-6.60, 6.57)	-6.35 (-21.01, 8.30)	0.44
	Trimester 2	-2.46 (-6.56, 1.63)	-7.24 (-16.50, 2.03)	0.36	-0.93 (-13.78, 11.92)	-10.93 (-25.19, 3.34)	0.30	-2.72 (-9.57, 4.14)	1.84 (-11.61, 15.29)	0.55
	Trimester 3	-2.64 (-6.84, 1.56)	-5.35 (-15.00, 4.30)	0.61	-2.83 (-16.45, 10.79)	-7.48 (-21.73, 6.78)	0.64	-2.76 (-9.49, 3.96)	6.48 (-6.98, 19.94)	0.23
	Pregnancy	-1.22 (-5.06, 2.61)	-6.93 (-15.90, 2.04)	0.25	-2.57 (-13.58, 8.45)	-9.47 (-22.98, 4.05)	0.43	-1.18 (-7.14, 4.78)	7.23 (-4.60, 19.05)	0.21
	Year 1	-1.90 (-5.54, 1.74)	-5.76 (-14.69, 3.17)	0.43	-2.92 (-14.08, 8.24)	-5.77 (-22.11, 10.58)	0.78	-0.88 (-6.04, 4.28)	6.92 (-4.36, 18.21)	0.22
	Year 2	-3.40 (-7.02, 0.21)	-9.87 (-18.50, -1.24)	0.18	-2.31 (-13.39, 8.78)	-3.70 (-18.68, 11.29)	0.88	-2.22 (-7.57, 3.13)	1.61 (-11.90, 15.12)	0.61
Any Flea/Tick Control Product	Pre-pregnancy	-0.80 (-5.01, 3.41)	**-15.82 (-25.32, -6.33)**	**<0.01**	-7.09 (-16.31, 2.13)	-4.89 (-19.42, 9.63)	0.80	0.67 (-2.53, 3.87)	0.44 (-5.93, 6.81)	0.95
	Trimester 1	0.50 (-3.65, 4.66)	**-14.29 (-23.30, -5.28)**	**<0.01**	-6.79 (-15.62, 2.04)	-4.88 (-19.40, 9.64)	0.83	-0.33 (-3.48, 2.83)	2.65 (-3.61, 8.91)	0.40
	Trimester 2	0.02 (-4.12, 4.15)	**-11.17 (-20.01, -2.32)**	**0.02**	-6.72 (-15.95, 2.50)	-4.91 (-19.44, 9.63)	0.84	-0.30 (-3.53, 2.94)	-0.12 (-6.38, 6.14)	0.96
	Trimester 3	-0.46 (-4.69, 3.76)	**-13.73 (-22.48, -4.98)**	**<0.01**	-7.05 (-16.94, 2.85)	-1.01 (-15.58, 13.55)	0.50	1.03 (-2.18, 4.25)	1.88 (-4.32, 8.09)	0.81
	Pregnancy	0.54 (-3.42, 4.50)	**-14.35 (-22.84, -5.85)**	**<0.01**	-6.02 (-14.73, 2.68)	-1.59 (-15.46, 12.28)	0.59	-0.17 (-3.10, 2.77)	0.83 (-5.11, 6.77)	0.77
	Year 1	-0.25 (-4.16, 3.66)	**-14.40 (-22.90, -5.89)**	**<0.01**	-6.77 (-15.20, 1.67)	-3.98 (-17.73, 9.76)	0.73	0.77 (-2.03, 3.58)	3.74 (-2.01, 9.49)	0.36
	Year 2	-0.81 (-4.73, 3.11)	**-9.43 (-17.79, -1.08)**	0.07	-5.54 (-14.23, 3.14)	-2.46 (-16.13, 11.21)	0.71	1.14 (-1.63, 3.91)	1.91 (-4.00, 7.82)	0.82

Bold typeface indicates statistical significance at $P < 0.05$. Dashes indicate that associations could not be estimated due to small sample sizes. Abbreviations: ASD, autism spectrum disorder; MSEL, Mullen Scales of Early Learning; P_{int}, P-value for interaction; VABS, Vineland Adaptive Behavior Scales.

Supplemental Table S7. Beta (95% CI) for association of Flea/Tick insecticide with MSEL Subscale DQ.

	Application	Exposure Period	N (exp/unexp)	Visual Receptivity	Receptive Language	Expressive Language	Fine Motor
ASD	Flea/Tick Collar Use	Pre-pregnancy	30/737	-6.22 (-15.45, 3.01)	-4.06 (-13.73, 5.60)	-6.29 (-15.34, 2.76)	-4.52 (-12.15, 3.10)
		Trimester 1	29/738	-6.28 (-15.68, 3.13)	-4.41 (-14.25, 5.43)	-6.91 (-16.12, 2.31)	-5.68 (-13.44, 2.09)
		Trimester 2	32/736	-7.15 (-16.11, 1.80)	-8.58 (-17.96, 0.79)	-8.71 (-17.49, 0.07)	-7.15 (-14.54, 0.24)
		Trimester 3	28/732	-3.42 (-12.99, 6.14)	-5.83 (-15.83, 4.18)	-6.62 (-15.99, 2.74)	-4.60 (-12.51, 3.30)
		Pregnancy	37/726	-5.42 (-13.81, 2.97)	-6.75 (-15.52, 2.02)	-7.43 (-15.64, 0.78)	-5.66 (-12.58, 1.27)
		Year 1	33/733	-5.45 (-14.27, 3.37)	-5.55 (-14.78, 3.69)	-7.30 (-15.95, 1.35)	-4.71 (-12.00, 2.58)
		Year 2	36/731	**-8.94 (-17.40, -0.48)**	-8.36 (-17.24, 0.51)	**-10.60 (-18.90, -2.30)**	-6.86 (-13.86, 0.14)
	Flea/Tick Skin application	Pre-pregnancy	109/635	-2.62 (-7.79, 2.56)	-0.36 (-5.82, 5.11)	0.01 (-5.10, 5.13)	-1.20 (-5.48, 3.07)
		Trimester 1	120/627	-3.35 (-8.35, 1.65)	0.57 (-4.71, 5.84)	0.75 (-4.17, 5.67)	-0.87 (-4.99, 3.26)
		Trimester 2	122/624	-0.63 (-5.59, 4.33)	2.15 (-3.07, 7.37)	2.92 (-1.96, 7.79)	1.01 (-3.07, 5.09)
		Trimester 3	114/625	-1.25 (-6.35, 3.85)	0.23 (-5.14, 5.59)	0.30 (-4.71, 5.31)	-0.10 (-4.30, 4.10)
		Pregnancy	139/606	-2.47 (-7.19, 2.25)	0.17 (-4.81, 5.14)	0.88 (-3.77, 5.52)	-0.51 (-4.41, 3.38)
		Year 1	148/607	-2.29 (-6.92, 2.33)	0.22 (-4.66, 5.09)	0.19 (-4.35, 4.74)	-0.96 (-4.77, 2.85)
		Year 2	138/619	-0.53 (-5.24, 4.17)	1.52 (-3.44, 6.48)	1.71 (-2.91, 6.33)	0.35 (-3.53, 4.23)
	Flea/Tick Soap, Shampoo, or Powder	Pre-pregnancy	71/683	-3.87 (-10.00, 2.27)	-6.01 (-12.43, 0.41)	-5.27 (-11.32, 0.77)	-4.14 (-9.25, 0.96)
		Trimester 1	69/684	-3.31 (-9.53, 2.90)	-6.40 (-12.89, 0.10)	-4.20 (-10.32, 1.92)	-4.57 (-9.74, 0.59)
		Trimester 2	73/682	-5.51 (-11.56, 0.55)	**-8.28 (-14.61, -1.94)**	-5.70 (-11.68, 0.27)	**-5.27 (-10.30, -0.23)**
		Trimester 3	69/676	-4.83 (-11.05, 1.39)	**-9.29 (-15.80, -2.77)**	-5.98 (-12.12, 0.16)	**-5.39 (-10.58, -0.19)**
		Pregnancy	85/663	-2.52 (-8.22, 3.17)	**-6.26 (-12.22, -0.29)**	-3.47 (-9.09, 2.15)	-3.88 (-8.63, 0.86)
		Year 1	94/665	**-6.12 (-11.55, -0.70)**	**-8.80 (-14.47, -3.14)**	**-5.84 (-11.18, -0.49)**	-4.18 (-8.70, 0.33)
		Year 2	96/662	**-7.64 (-13.01, -2.26)**	**-10.54 (-16.16, -4.92)**	**-9.40 (-14.69, -4.12)**	**-6.88 (-11.36, -2.41)**
	Any Flea/Tick Control Product	Pre-pregnancy	162/615	-3.26 (-7.65, 1.12)	-3.08 (-7.68, 1.53)	-3.02 (-7.32, 1.29)	-3.06 (-6.70, 0.57)
		Trimester 1	173/604	-2.81 (-7.10, 1.47)	-1.77 (-6.28, 2.74)	-1.05 (-5.27, 3.16)	-2.21 (-5.77, 1.35)
		Trimester 2	177/601	-2.69 (-6.94, 1.56)	-2.16 (-6.63, 2.31)	-0.79 (-4.97, 3.39)	-1.53 (-5.06, 1.99)
		Trimester 3	168/604	-2.23 (-6.57, 2.10)	-3.76 (-8.31, 0.78)	-2.70 (-6.95, 1.55)	-2.14 (-5.74, 1.46)
		Pregnancy	200/576	-2.13 (-6.23, 1.97)	-2.54 (-6.85, 1.77)	-1.08 (-5.11, 2.95)	-1.98 (-5.38, 1.43)
		Year 1	206/572	-3.42 (-7.46, 0.63)	-2.63 (-6.88, 1.63)	-1.92 (-5.90, 2.06)	-2.05 (-5.41, 1.31)
		Year 2	207/572	-2.89 (-6.90, 1.12)	-2.39 (-6.62, 1.83)	-1.73 (-5.67, 2.22)	-2.05 (-5.38, 1.27)
DD	Flea/Tick Collar Use	Pre-pregnancy	5/177	**-25.05 (-46.71, -3.39)**	-14.87 (-32.38, 2.64)	-20.27 (-40.88, 0.35)	-16.96 (-34.41, 0.50)
		Trimester 1	5/177	**-25.05 (-46.71, -3.39)**	-14.87 (-32.38, 2.64)	-20.27 (-40.88, 0.35)	-16.96 (-34.41, 0.50)
		Trimester 2	5/177	-21.48 (-43.41, 0.45)	-9.14 (-26.88, 8.60)	-14.08 (-34.99, 6.82)	-14.45 (-32.11, 3.20)
		Trimester 3	5/170	-21.54 (-43.69, 0.61)	-9.00 (-26.81, 8.82)	-14.17 (-35.06, 6.72)	-14.56 (-32.38, 3.27)
		Pregnancy	6/174	**-21.22 (-41.25, -1.19)**	-9.11 (-25.34, 7.13)	-10.72 (-29.79, 8.36)	-13.04 (-29.23, 3.15)
		Year 1	7/178	-16.31 (-35.04, 2.42)	-5.32 (-20.47, 9.84)	-12.73 (-30.67, 5.21)	-11.60 (-26.71, 3.50)
		Year 2	6/179	-18.34 (-38.34, 1.66)	-9.16 (-25.31, 7.00)	**-20.44 (-39.48, -1.40)**	-14.35 (-30.46, 1.76)
	Flea/Tick Skin application	Pre-pregnancy	18/160	**-15.21 (-27.07, -3.35)**	**-9.71 (-19.31, -0.12)**	-7.73 (-19.16, 3.69)	-7.05 (-16.76, 2.65)
		Trimester 1	17/161	**-16.13 (-28.22, -4.03)**	**-11.70 (-21.45, -1.95)**	-7.89 (-19.56, 3.78)	-8.81 (-18.69, 1.07)
		Trimester 2	16/161	**-17.88 (-30.47, -5.29)**	**-13.55 (-23.67, -3.44)**	-10.43 (-22.51, 1.64)	-9.56 (-19.86, 0.75)
		Trimester 3	13/156	**-14.50 (-28.70, -0.30)**	**-12.00 (-23.30, -0.70)**	-8.27 (-21.68, 5.14)	-7.65 (-19.23, 3.93)
		Pregnancy	18/156	**-16.99 (-28.83, -5.16)**	**-11.90 (-21.45, -2.36)**	-8.86 (-20.21, 2.49)	-9.25 (-18.98, 0.48)
		Year 1	26/155	**-11.94 (-22.23, -1.65)**	-6.42 (-14.74, 1.90)	-3.22 (-13.15, 6.71)	-5.59 (-13.99, 2.81)
		Year 2	26/157	-8.96 (-19.28, 1.36)	-2.82 (-11.17, 5.53)	-2.30 (-12.22, 7.63)	-4.58 (-12.94, 3.79)

	Flea/Tick Soap, Shampoo, or Powder	Pre-pregnancy	20/161	-9.96 (-21.35, 1.44)	-3.17 (-12.39, 6.05)	-5.91 (-16.79, 4.97)	-4.01 (-13.25, 5.23)
		Trimester 1	18/163	-4.96 (-17.07, 7.14)	1.26 (-8.48, 11.01)	2.96 (-8.56, 14.48)	-1.33 (-11.10, 8.43)
		Trimester 2	15/166	-4.72 (-17.99, 8.56)	0.58 (-10.10, 11.26)	2.22 (-10.41, 14.84)	-0.96 (-11.67, 9.74)
		Trimester 3	14/159	-4.69 (-18.42, 9.03)	0.02 (-10.95, 10.99)	2.10 (-10.83, 15.04)	0.19 (-10.90, 11.28)
		Pregnancy	19/160	-3.81 (-15.67, 8.05)	1.06 (-8.49, 10.61)	3.97 (-7.35, 15.28)	0.31 (-9.30, 9.91)
		Year 1	16/166	-7.41 (-20.16, 5.33)	0.99 (-9.28, 11.25)	1.95 (-10.28, 14.18)	-1.47 (-11.78, 8.84)
		Year 2	17/166	-9.71 (-22.09, 2.66)	-0.14 (-10.13, 9.86)	0.35 (-11.54, 12.24)	-3.05 (-13.06, 6.96)
	Any Flea/Tick Control Product	Pre-pregnancy	28/156	**-10.71 (-20.52, -0.90)**	-4.91 (-12.86, 3.04)	-4.14 (-13.58, 5.29)	-5.82 (-13.77, 2.13)
		Trimester 1	30/154	**-11.04 (-20.52, -1.56)**	-4.81 (-12.50, 2.89)	-1.52 (-10.67, 7.63)	-6.50 (-14.18, 1.18)
		Trimester 2	28/156	**-11.38 (-21.17, -1.58)**	-4.98 (-12.93, 2.97)	-1.60 (-11.05, 7.85)	-6.51 (-14.44, 1.43)
		Trimester 3	25/152	-10.34 (-20.80, 0.11)	-4.48 (-12.88, 3.93)	-1.11 (-11.06, 8.83)	-5.36 (-13.82, 3.10)
		Pregnancy	32/152	**-9.85 (-19.11, -0.60)**	-3.94 (-11.44, 3.57)	0.56 (-8.35, 9.48)	-4.87 (-12.38, 2.63)
		Year 1	35/150	**-10.42 (-19.46, -1.38)**	-3.74 (-11.09, 3.61)	-1.57 (-10.33, 7.19)	-5.53 (-12.87, 1.81)
		Year 2	33/152	-8.81 (-18.00, 0.39)	-2.31 (-9.77, 5.14)	-1.47 (-10.34, 7.40)	-4.51 (-11.96, 2.93)
TD	Flea/Tick Collar Use	Pre-pregnancy	14/500	-3.35 (-12.65, 5.94)	-2.80 (-11.93, 6.33)	-0.83 (-10.39, 8.74)	-0.42 (-8.29, 7.46)
		Trimester 1	16/498	-4.10 (-12.78, 4.58)	-1.49 (-10.01, 7.03)	-3.65 (-12.56, 5.27)	-1.27 (-8.62, 6.08)
		Trimester 2	17/495	-1.33 (-9.75, 7.08)	-2.46 (-10.71, 5.79)	-1.34 (-9.99, 7.32)	-2.14 (-9.28, 4.99)
		Trimester 3	13/498	-5.65 (-15.23, 3.93)	-0.20 (-9.65, 9.24)	-0.04 (-9.95, 9.86)	0.72 (-7.39, 8.83)
		Pregnancy	22/490	-3.39 (-10.86, 4.08)	-3.84 (-11.17, 3.48)	-3.75 (-11.43, 3.93)	-2.78 (-9.11, 3.55)
		Year 1	17/495	-4.37 (-12.84, 4.09)	1.61 (-6.71, 9.92)	1.25 (-7.45, 9.94)	0.97 (-6.20, 8.14)
		Year 2	15/497	-4.61 (-13.59, 4.36)	-3.04 (-11.85, 5.76)	-4.58 (-13.76, 4.60)	-2.85 (-10.43, 4.73)
	Flea/Tick Skin application	Pre-pregnancy	75/425	4.23 (-0.17, 8.64)	1.87 (-2.48, 6.22)	1.85 (-2.70, 6.41)	1.12 (-2.59, 4.83)
		Trimester 1	79/420	2.22 (-2.16, 6.59)	1.79 (-2.50, 6.09)	3.33 (-1.17, 7.83)	-0.09 (-3.76, 3.58)
		Trimester 2	74/426	3.36 (-1.08, 7.81)	-0.84 (-5.22, 3.53)	2.08 (-2.50, 6.67)	0.32 (-3.42, 4.06)
		Trimester 3	77/422	4.23 (-0.12, 8.58)	2.34 (-1.95, 6.64)	2.74 (-1.77, 7.24)	1.54 (-2.11, 5.19)
		Pregnancy	95/405	2.70 (-1.35, 6.74)	1.28 (-2.70, 5.25)	2.92 (-1.25, 7.08)	1.01 (-2.38, 4.41)
		Year 1	115/394	3.68 (-0.05, 7.41)	2.68 (-0.98, 6.35)	3.39 (-0.43, 7.22)	-0.19 (-3.34, 2.96)
		Year 2	118/390	**4.10 (0.43, 7.77)**	0.89 (-2.73, 4.52)	2.56 (-1.22, 6.33)	0.99 (-2.12, 4.09)
	Flea/Tick Soap, Shampoo, or Powder	Pre-pregnancy	32/465	1.56 (-4.70, 7.83)	-4.08 (-10.20, 2.04)	-2.06 (-8.47, 4.35)	-3.94 (-9.21, 1.33)
		Trimester 1	30/466	-1.51 (-7.97, 4.95)	-2.75 (-9.06, 3.57)	-1.96 (-8.58, 4.65)	-4.72 (-10.16, 0.72)
		Trimester 2	29/469	-1.64 (-8.21, 4.94)	-5.55 (-11.99, 0.90)	-2.19 (-8.96, 4.57)	-4.37 (-9.88, 1.15)
		Trimester 3	30/465	-0.77 (-7.24, 5.71)	-4.39 (-10.75, 1.97)	-3.12 (-9.78, 3.54)	-3.97 (-9.38, 1.44)
		Pregnancy	39/458	-1.13 (-6.88, 4.62)	-3.30 (-8.94, 2.33)	-2.84 (-8.74, 3.05)	-3.16 (-7.98, 1.65)
		Year 1	52/454	1.95 (-3.11, 7.01)	-1.48 (-6.44, 3.48)	-2.23 (-7.40, 2.94)	-1.35 (-5.62, 2.92)
		Year 2	46/459	4.35 (-0.97, 9.68)	-2.67 (-7.90, 2.57)	-4.65 (-10.08, 0.79)	-2.97 (-7.47, 1.53)
	Any Flea/Tick Control Product	Pre-pregnancy	99/419	2.74 (-1.13, 6.60)	-0.13 (-3.93, 3.67)	0.63 (-3.37, 4.64)	-0.59 (-3.86, 2.68)
		Trimester 1	105/413	0.36 (-3.45, 4.18)	0.26 (-3.48, 4.01)	1.24 (-2.70, 5.19)	-0.81 (-4.04, 2.41)
		Trimester 2	98/419	1.68 (-2.21, 5.58)	-1.64 (-5.46, 2.19)	0.63 (-3.41, 4.66)	-1.26 (-4.56, 2.03)
		Trimester 3	100/416	1.93 (-1.93, 5.78)	1.36 (-2.44, 5.16)	1.66 (-2.35, 5.66)	0.44 (-2.81, 3.69)
		Pregnancy	126/391	0.76 (-2.81, 4.33)	-0.75 (-4.26, 2.75)	0.60 (-3.10, 4.29)	-0.47 (-3.49, 2.55)
		Year 1	145/373	3.06 (-0.38, 6.49)	1.06 (-2.32, 4.44)	2.12 (-1.44, 5.68)	-1.07 (-3.97, 1.84)
		Year 2	146/371	**3.82 (0.44, 7.19)**	-0.14 (-3.48, 3.21)	1.42 (-2.10, 4.93)	-0.64 (-3.51, 2.23)

Bold typeface indicates statistical significance at $P < 0.05$. Associations for Flea/Tick collars among DD were not assessed due to small sample sizes. Abbreviations: ASD, autism spectrum disorder; DD, developmental delay; DQ, developmental quotient; MSEL, Mullen Scales of Early Learning; TD, typically developing

Supplemental Table S8. Beta (95% CI) for association of Flea/Tick insecticide with VABS Subscale DQ**.**

	Application	Exposure Period	N (exp/unexp)	Communication	Daily Living Skills	Socialization	Fine Motor Skills
ASD	Flea/Tick Collar Use	Pre-pregnancy	30/739	-7.84 (-15.98, 0.31)	-2.21 (-7.37, 2.94)	-4.83 (-11.65, 1.98)	-3.15 (-10.21, 3.91)
		Trimester 1	29/740	-7.21 (-15.51, 1.09)	-1.87 (-7.12, 3.38)	-3.45 (-10.40, 3.49)	-3.76 (-10.95, 3.43)
		Trimester 2	32/738	**-7.96 (-15.86, -0.06)**	-3.23 (-8.23, 1.77)	-1.43 (-8.05, 5.19)	-5.29 (-12.13, 1.56)
		Trimester 3	28/734	-6.17 (-14.61, 2.27)	-3.80 (-9.13, 1.54)	-0.97 (-8.03, 6.08)	**-8.08 (-15.37, -0.79)**
		Pregnancy	37/728	**-7.75 (-15.15, -0.36)**	-2.63 (-7.32, 2.05)	-2.28 (-8.47, 3.92)	-5.73 (-12.14, 0.68)
		Year 1	33/735	-3.34 (-11.14, 4.45)	-1.09 (-6.01, 3.83)	-1.30 (-7.82, 5.21)	-5.89 (-12.62, 0.84)
		Year 2	36/733	-3.22 (-10.71, 4.28)	-2.01 (-6.74, 2.72)	0.38 (-5.89, 6.66)	-5.39 (-11.86, 1.08)
	Flea/Tick Skin application	Pre-pregnancy	108/638	-1.14 (-5.73, 3.44)	0.77 (-2.09, 3.64)	**4.04 (0.20, 7.88)**	0.64 (-3.32, 4.61)
		Trimester 1	120/629	-1.49 (-5.93, 2.95)	1.69 (-1.06, 4.44)	**4.15 (0.45, 7.84)**	1.38 (-2.43, 5.18)
		Trimester 2	121/627	0.39 (-3.98, 4.77)	1.30 (-1.44, 4.03)	**4.54 (0.87, 8.21)**	0.89 (-2.89, 4.67)
		Trimester 3	113/628	-0.65 (-5.14, 3.84)	1.90 (-0.92, 4.72)	**4.25 (0.48, 8.02)**	2.29 (-1.59, 6.17)
		Pregnancy	139/608	-1.41 (-5.57, 2.76)	1.20 (-1.40, 3.81)	3.29 (-0.21, 6.79)	0.97 (-2.63, 4.57)
		Year 1	147/610	-0.37 (-4.44, 3.70)	0.88 (-1.70, 3.47)	**3.49 (0.07, 6.91)**	0.12 (-3.41, 3.65)
		Year 2	138/622	0.29 (-3.85, 4.43)	1.26 (-1.37, 3.89)	**3.72 (0.25, 7.20)**	-0.26 (-3.86, 3.33)
	Flea/Tick Soap, Shampoo, or Powder	Pre-pregnancy	72/685	-3.60 (-9.05, 1.86)	-3.10 (-6.53, 0.33)	-1.74 (-6.24, 2.76)	-2.43 (-7.08, 2.22)
		Trimester 1	70/686	-2.69 (-8.21, 2.84)	-2.84 (-6.31, 0.63)	-1.35 (-5.90, 3.20)	-1.91 (-6.62, 2.79)
		Trimester 2	74/684	-4.52 (-9.91, 0.87)	-3.15 (-6.54, 0.24)	-1.89 (-6.33, 2.56)	-3.31 (-7.90, 1.28)
		Trimester 3	70/678	-4.07 (-9.62, 1.48)	-2.65 (-6.15, 0.84)	-1.71 (-6.28, 2.87)	-2.48 (-7.21, 2.25)
		Pregnancy	86/665	-1.42 (-6.50, 3.67)	-2.27 (-5.46, 0.93)	-1.01 (-5.19, 3.18)	-2.76 (-7.09, 1.57)
		Year 1	95/667	-3.36 (-8.18, 1.47)	-1.83 (-4.87, 1.20)	-1.84 (-5.84, 2.15)	-1.69 (-5.82, 2.44)
		Year 2	97/664	**-5.83 (-10.62, -1.04)**	**-3.56 (-6.57, -0.56)**	-3.21 (-7.18, 0.76)	-3.88 (-7.98, 0.21)
	Any Flea/Tick Control Product	Pre-pregnancy	162/618	-2.97 (-6.86, 0.93)	-0.84 (-3.29, 1.61)	0.78 (-2.47, 4.04)	-1.37 (-4.72, 1.99)
		Trimester 1	174/606	-2.15 (-5.95, 1.66)	-0.01 (-2.40, 2.39)	1.65 (-1.53, 4.82)	-0.44 (-3.72, 2.84)
		Trimester 2	177/604	-1.90 (-5.67, 1.87)	-0.23 (-2.61, 2.14)	1.49 (-1.66, 4.65)	-1.06 (-4.31, 2.19)
		Trimester 3	168/607	-2.55 (-6.39, 1.30)	-0.31 (-2.73, 2.12)	1.13 (-2.08, 4.35)	-1.17 (-4.48, 2.15)
		Pregnancy	201/578	-1.74 (-5.38, 1.90)	-0.29 (-2.58, 2.00)	0.91 (-2.13, 3.95)	-1.47 (-4.61, 1.67)
		Year 1	206/575	-1.14 (-4.73, 2.46)	-0.06 (-2.33, 2.20)	1.33 (-1.67, 4.33)	-1.73 (-4.82, 1.36)
		Year 2	208/574	-1.43 (-4.99, 2.13)	-0.23 (-2.47, 2.01)	1.45 (-1.52, 4.43)	-2.01 (-5.08, 1.05)
DD	Flea/Tick Collar Use	Pre-pregnancy	5/175	-16.27 (-34.23, 1.69)	-14.05 (-31.19, 3.09)	-12.14 (-32.28, 7.99)	-13.50 (-32.43, 5.43)
		Trimester 1	5/175	-16.27 (-34.23, 1.69)	-14.05 (-31.19, 3.09)	-12.14 (-32.28, 7.99)	-13.50 (-32.43, 5.43)
		Trimester 2	5/175	-9.67 (-27.89, 8.55)	-7.26 (-24.63, 10.12)	-9.75 (-30.08, 10.58)	-8.01 (-27.17, 11.14)
		Trimester 3	5/168	-9.40 (-27.94, 9.13)	-6.76 (-23.69, 10.16)	-10.24 (-30.82, 10.33)	-8.42 (-27.61, 10.78)
		Pregnancy	6/172	-8.76 (-25.48, 7.96)	-7.46 (-23.42, 8.49)	-10.99 (-29.68, 7.70)	-11.15 (-28.70, 6.40)
		Year 1	7/176	-8.50 (-24.33, 7.33)	-5.52 (-20.35, 9.31)	-8.06 (-25.41, 9.30)	-5.03 (-21.35, 11.29)
		Year 2	6/177	-14.42 (-31.26, 2.41)	-10.81 (-26.60, 4.98)	-8.54 (-27.09, 10.01)	-6.08 (-23.52, 11.36)
	Flea/Tick Skin application	Pre-pregnancy	18/158	-6.91 (-16.79, 2.96)	-7.62 (-17.01, 1.77)	**-16.62 (-27.26, -5.97)**	-10.32 (-20.69, 0.05)
		Trimester 1	17/159	-6.75 (-16.84, 3.34)	-7.48 (-17.08, 2.12)	**-16.64 (-27.53, -5.76)**	-9.83 (-20.44, 0.78)
		Trimester 2	16/159	-8.08 (-18.54, 2.39)	-9.32 (-19.26, 0.62)	**-18.16 (-29.49, -6.84)**	-10.39 (-21.46, 0.68)
		Trimester 3	13/154	-5.86 (-17.68, 5.97)	-8.48 (-19.21, 2.25)	**-18.61 (-31.31, -5.90)**	-10.42 (-22.60, 1.77)
		Pregnancy	18/154	-7.82 (-17.71, 2.07)	-7.82 (-17.25, 1.61)	**-17.52 (-28.23, -6.81)**	-10.17 (-20.55, 0.22)
		Year 1	26/153	-0.34 (-9.06, 8.37)	-1.74 (-9.90, 6.43)	**-9.88 (-19.26, -0.50)**	-2.66 (-11.62, 6.31)
		Year 2	26/155	2.17 (-6.53, 10.87)	-1.80 (-9.96, 6.35)	-9.14 (-18.51, 0.22)	-1.75 (-10.74, 7.25)

	Flea/Tick Soap, Shampoo, or Powder	Pre-pregnancy	20/159	-7.61 (-17.05, 1.82)	-6.75 (-15.70, 2.19)	**-12.40 (-22.80, -2.00)**	-9.25 (-19.13, 0.63)
		Trimester 1	18/161	-1.44 (-11.46, 8.59)	-6.45 (-15.90, 3.00)	-9.57 (-20.62, 1.49)	-7.86 (-18.33, 2.60)
		Trimester 2	15/164	-2.02 (-13.01, 8.97)	-7.51 (-17.86, 2.83)	-8.53 (-20.67, 3.61)	-9.72 (-21.16, 1.72)
		Trimester 3	14/157	-1.66 (-13.11, 9.80)	-8.00 (-18.32, 2.32)	-9.20 (-21.78, 3.39)	-8.47 (-20.15, 3.21)
		Pregnancy	19/158	-1.65 (-11.51, 8.21)	-6.91 (-16.19, 2.38)	-10.23 (-21.05, 0.59)	-6.64 (-16.82, 3.53)
		Year 1	16/164	3.24 (-7.54, 14.02)	-5.34 (-15.37, 4.68)	-6.75 (-18.52, 5.03)	-5.54 (-16.54, 5.46)
		Year 2	17/164	4.00 (-6.46, 14.45)	-3.71 (-13.45, 6.04)	-5.87 (-17.33, 5.58)	-6.53 (-17.28, 4.21)
	Any Flea/Tick Control Product	Pre-pregnancy	28/154	-5.55 (-13.71, 2.61)	**-7.94 (-15.65, -0.24)**	**-12.16 (-21.11, -3.22)**	**-9.84 (-18.31, -1.37)**
		Trimester 1	30/152	-3.44 (-11.37, 4.48)	**-8.32 (-15.77, -0.87)**	**-10.42 (-19.11, -1.72)**	**-9.98 (-18.17, -1.79)**
		Trimester 2	28/154	-2.72 (-10.91, 5.47)	**-7.75 (-15.46, -0.04)**	**-10.28 (-19.27, -1.28)**	**-9.38 (-17.86, -0.90)**
		Trimester 3	25/150	-1.80 (-10.57, 6.97)	**-7.97 (-15.87, -0.08)**	**-10.87 (-20.45, -1.29)**	**-9.49 (-18.44, -0.54)**
		Pregnancy	32/150	-2.36 (-10.09, 5.36)	**-7.32 (-14.59, -0.05)**	**-10.57 (-19.02, -2.11)**	**-8.76 (-16.76, -0.76)**
		Year 1	35/148	0.48 (-7.24, 8.20)	-2.88 (-10.08, 4.33)	-7.21 (-15.59, 1.17)	-4.80 (-12.70, 3.11)
		Year 2	33/150	2.68 (-5.13, 10.48)	-2.17 (-9.47, 5.13)	-6.50 (-15.00, 2.01)	-4.06 (-12.08, 3.96)
TD	Flea/Tick Collar Use	Pre-pregnancy	14/500	-0.69 (-12.00, 10.63)	6.26 (-4.31, 16.83)	2.83 (-9.97, 15.62)	8.47 (-1.98, 18.92)
		Trimester 1	16/498	-3.31 (-13.89, 7.28)	7.34 (-2.54, 17.22)	2.45 (-9.50, 14.40)	4.29 (-5.48, 14.07)
		Trimester 2	17/495	3.30 (-6.94, 13.55)	6.55 (-3.02, 16.13)	5.12 (-6.43, 16.68)	6.03 (-3.44, 15.49)
		Trimester 3	13/498	-4.65 (-16.38, 7.07)	4.76 (-6.21, 15.73)	5.52 (-7.71, 18.75)	3.28 (-7.56, 14.13)
		Pregnancy	22/490	-1.51 (-10.63, 7.60)	5.22 (-3.29, 13.72)	7.72 (-2.54, 17.98)	5.27 (-3.14, 13.69)
		Year 1	17/495	-2.20 (-12.49, 8.09)	5.07 (-4.56, 14.69)	6.04 (-5.58, 17.67)	3.65 (-5.88, 13.18)
		Year 2	15/497	-7.65 (-18.49, 3.18)	1.18 (-9.03, 11.40)	-0.82 (-13.13, 11.50)	2.64 (-7.46, 12.74)
	Flea/Tick Skin application	Pre-pregnancy	75/425	2.96 (-2.40, 8.33)	3.16 (-1.82, 8.14)	3.43 (-2.62, 9.48)	3.45 (-1.47, 8.36)
		Trimester 1	79/420	2.35 (-2.96, 7.67)	1.08 (-3.86, 6.02)	-0.20 (-6.20, 5.80)	2.34 (-2.53, 7.21)
		Trimester 2	74/426	1.99 (-3.42, 7.40)	1.15 (-3.88, 6.17)	0.06 (-6.04, 6.17)	0.50 (-4.46, 5.46)
		Trimester 3	77/422	2.84 (-2.47, 8.16)	2.03 (-2.91, 6.97)	0.98 (-5.02, 6.99)	0.79 (-4.09, 5.67)
		Pregnancy	95/405	3.04 (-1.88, 7.96)	0.32 (-4.25, 4.89)	0.13 (-5.42, 5.69)	1.49 (-3.02, 6.00)
		Year 1	115/394	2.45 (-2.09, 6.98)	-0.60 (-4.82, 3.62)	-3.02 (-8.13, 2.08)	-0.34 (-4.51, 3.84)
		Year 2	118/390	1.12 (-3.34, 5.58)	-1.76 (-5.93, 2.40)	-1.26 (-6.30, 3.77)	1.07 (-3.05, 5.19)
	Flea/Tick Soap, Shampoo, or Powder	Pre-pregnancy	32/465	-0.32 (-7.95, 7.32)	1.16 (-6.00, 8.32)	0.54 (-8.18, 9.25)	-1.55 (-8.58, 5.48)
		Trimester 1	30/466	0.02 (-7.84, 7.87)	-0.74 (-8.11, 6.62)	-2.16 (-11.13, 6.81)	-1.66 (-8.90, 5.58)
		Trimester 2	29/469	-1.60 (-9.59, 6.40)	-1.12 (-8.60, 6.36)	-1.29 (-10.40, 7.82)	-3.54 (-10.88, 3.81)
		Trimester 3	30/465	-3.18 (-11.05, 4.69)	-0.18 (-7.57, 7.20)	2.40 (-6.59, 11.40)	-2.33 (-9.57, 4.92)
		Pregnancy	39/458	0.77 (-6.20, 7.74)	1.32 (-5.20, 7.84)	2.09 (-5.86, 10.05)	-0.33 (-6.74, 6.09)
		Year 1	52/454	1.75 (-4.40, 7.90)	-0.56 (-6.32, 5.21)	-0.14 (-7.11, 6.82)	-0.06 (-5.71, 5.59)
		Year 2	46/459	-2.62 (-9.10, 3.85)	-2.88 (-8.97, 3.21)	-1.18 (-8.52, 6.16)	-3.25 (-9.22, 2.72)
	Any Flea/Tick Control Product	Pre-pregnancy	99/419	0.91 (-3.80, 5.61)	2.60 (-1.79, 6.99)	1.28 (-4.04, 6.60)	1.29 (-3.05, 5.63)
		Trimester 1	105/413	1.05 (-3.58, 5.69)	1.78 (-2.55, 6.11)	-0.06 (-5.30, 5.18)	1.35 (-2.93, 5.63)
		Trimester 2	98/419	1.18 (-3.55, 5.91)	1.34 (-3.08, 5.76)	-0.22 (-5.58, 5.13)	0.22 (-4.15, 4.59)
		Trimester 3	100/416	1.37 (-3.33, 6.06)	1.52 (-2.87, 5.91)	1.23 (-4.09, 6.55)	0.11 (-4.24, 4.45)
		Pregnancy	126/391	1.27 (-3.06, 5.60)	1.19 (-2.86, 5.24)	1.18 (-3.73, 6.08)	1.29 (-2.72, 5.30)
		Year 1	145/373	1.71 (-2.47, 5.89)	-0.96 (-4.87, 2.95)	-2.39 (-7.12, 2.34)	-1.93 (-5.79, 1.93)
		Year 2	146/371	-0.03 (-4.15, 4.08)	-1.45 (-5.32, 2.42)	-1.33 (-5.99, 3.33)	-0.63 (-4.46, 3.19)

Bold typeface indicates statistical significance at *P*< 0.05. Associations for Flea/Tick collars among DD were not assessed due to small sample sizes. Abbreviations: ASD, autism spectrum disorder; DD, developmental delay; DQ, developmental quotient; MSEL, Mullen Scales of Early Learning; TD, typically developing

Supplemental Table S9. Beta (95% CI) for associations of frequency of each insecticide with MSEL Composite DQ**.**

Application	Exposure Period	Frequency	ASD N Exp/unexp	ASD Beta (95% CI)	DD N Exp/unexp	DD Beta (95% CI)	TD N Exp/unexp	TD Beta (95% CI)
Indoor Professionally Applied Insecticide	Pre-pregnancy	1-2 mos	10/734	7.05 (-6.81, 20.90)	5/176	-	7/498	-5.36 (-15.10, 4.37)
		3 mos	24/734	7.63 (-1.39, 16.65)	1/176	-	5/498	-7.08 (-18.49, 4.34)
	Trimester 1	1-2 mos	8/728	8.62 (-6.82, 24.05)	5/176	-	6/498	-2.77 (-13.23, 7.70)
		3 mos	26/728	6.80 (-1.92, 15.51)	1/176	-	5/498	-7.02 (-18.45, 4.41)
	Trimester 2	1-2 mos	18/717	8.98 (-1.33, 19.29)	4/177	-	10/494	-3.86 (-12.05, 4.34)
		3 mos	26/717	1.44 (-7.28, 10.16)	1/177	-	6/494	-1.41 (-11.87, 9.04)
	Trimester 3	1-2 mos	16/714	9.42 (-1.54, 20.37)	1/173	-	11/492	0.39 (-7.43, 8.21)
		3 mos	26/714	2.02 (-6.72, 10.75)	1/173	-	5/492	-6.89 (-18.32, 4.53)
	Pregnancy	1-5 mos	26/703	**14.44 (5.84, 23.04)**	5/176	-	13/490	-1.62 (-8.81, 5.57)
		6-9 mos	28/703	2.01 (-6.35, 10.36)	1/176	-	6/490	-5.21 (-15.69, 5.27)
Indoor Non-professionally Applied Insecticide	Pre-pregnancy	1-2 mos	34/665	-3.69 (-11.39, 4.00)	12/152	-1.59 (-13.21, 10.03)	30/448	-0.20 (-5.03, 4.63)
		3 mos	68/665	2.47 (-3.12, 8.07)	15/152	**-10.93 (-21.68, -0.18)**	33/448	-0.82 (-5.40, 3.76)
	Trimester 1	1-2 mos	41/655	2.33 (-4.67, 9.33)	13/147	0.83 (-10.13, 11.80)	31/448	-2.85 (-7.60, 1.90)
		3 mos	71/655	2.81 (-2.65, 8.28)	19/147	**-12.54 (-22.09, -2.99)**	32/448	-1.06 (-5.71, 3.60)
	Trimester 2	1-2 mos	54/640	0.24 (-5.96, 6.44)	19/145	3.63 (-5.73, 12.99)	42/437	0.59 (-3.54, 4.72)
		3 mos	72/640	2.86 (-2.60, 8.31)	15/145	**-11.43 (-21.99, -0.88)**	32/437	-0.21 (-4.88, 4.45)
	Trimester 3	1-2 mos	37/658	3.01 (-4.35, 10.37)	11/144	-1.79 (-13.92, 10.35)	27/450	-2.85 (-7.98, 2.27)
		3 mos	66/658	3.41 (-2.26, 9.09)	19/144	**-10.91 (-20.59, -1.24)**	32/450	-0.31 (-4.97, 4.34)
	Pregnancy	1-5 mos	106/595	1.64 (-3.01, 6.30)	32/130	-1.01 (-8.60, 6.58)	78/403	-1.50 (-4.70, 1.71)
		6-9 mos	63/595	3.49 (-2.31, 9.29)	17/130	**-10.02 (-20.01, -0.03)**	29/403	0.28 (-4.62, 5.19)
Any Indoor Insecticide	Pre-pregnancy	1-2 mos	42/643	-1.73 (-8.69, 5.22)	15/148	-1.39 (-11.72, 8.93)	35/441	-0.86 (-5.36, 3.64)
		3 mos	88/643	3.59 (-1.40, 8.59)	16/148	**-11.01 (-21.40, -0.61)**	37/441	-1.25 (-5.60, 3.10)
	Trimester 1	1-2 mos	44/635	3.14 (-3.64, 9.92)	17/143	0.50 (-9.19, 10.19)	36/441	-3.13 (-7.57, 1.30)
		3 mos	93/635	3.76 (-1.12, 8.63)	20/143	**-12.55 (-21.82, -3.28)**	36/441	-1.47 (-5.88, 2.94)
	Trimester 2	1-2 mos	65/613	3.69 (-2.01, 9.39)	22/141	3.19 (-5.67, 12.05)	46/429	1.13 (-2.83, 5.10)
		3 mos	94/613	2.17 (-2.72, 7.06)	16/141	**-11.46 (-21.69, -1.24)**	38/429	-1.87 (-6.18, 2.44)
	Trimester 3	1-2 mos	48/631	5.55 (-0.96, 12.06)	12/142	-0.34 (-11.84, 11.17)	34/440	-3.38 (-7.95, 1.19)
		3 mos	87/631	3.16 (-1.87, 8.20)	20/142	**-10.92 (-20.34, -1.50)**	37/440	-1.34 (-5.69, 3.00)
	Pregnancy	1-5 mos	121/565	**5.31 (0.90, 9.71)**	35/129	-0.76 (-8.04, 6.53)	82/396	-1.73 (-4.86, 1.40)
		6-9 mos	86/565	3.20 (-1.87, 8.27)	18/129	**-10.07 (-19.78, -0.37)**	35/396	-0.79 (-5.28, 3.71)
Outdoor Non-professionally Applied Insecticide	Pre-pregnancy	1-2 mos	32/689	-4.76 (-12.66, 3.13)	6/172	0.19 (-15.95, 16.33)	34/459	0.61 (-3.99, 5.21)
		3 mos	44/689	0.28 (-6.50, 7.07)	5/172	-2.66 (-20.15, 14.82)	14/459	-2.28 (-9.27, 4.71)
	Trimester 1	1-2 mos	35/680	2.76 (-4.83, 10.34)	9/167	-0.68 (-14.03, 12.67)	38/457	-0.76 (-5.11, 3.58)
		3 mos	50/680	1.60 (-4.78, 7.98)	7/167	-1.77 (-16.75, 13.22)	12/457	-1.21 (-8.77, 6.35)
	Trimester 2	1-2 mos	41/669	-5.51 (-12.54, 1.51)	12/165	2.11 (-9.60, 13.81)	40/450	1.21 (-3.01, 5.42)
		3 mos	54/669	3.01 (-3.14, 9.17)	6/165	-7.97 (-24.17, 8.23)	18/450	-0.38 (-6.56, 5.79)
	Trimester 3	1-2 mos	38/677	-1.82 (-9.13, 5.49)	11/156	-2.66 (-14.91, 9.59)	33/458	-3.48 (-8.13, 1.17)
		3 mos	43/677	3.20 (-3.67, 10.08)	9/156	-6.54 (-20.04, 6.97)	16/458	0.76 (-5.76, 7.27)
	Pregnancy	1-5 mos	89/631	-3.90 (-8.89, 1.10)	21/151	2.33 (-6.94, 11.61)	85/413	-1.24 (-4.33, 1.85)
		6-9 mos	42/631	3.89 (-3.05, 10.83)	7/151	-7.95 (-22.99, 7.09)	11/413	0.26 (-7.58, 8.09)

Any Outdoor Insecticide	Pre-pregnancy	1-2 mos	42/634	-3.94 (-10.94, 3.06)	8/161	-1.83 (-15.85, 12.20)	49/421	0.74 (-3.16, 4.63)
		3 mos	89/634	3.12 (-1.89, 8.12)	14/161	-1.05 (-12.06, 9.95)	37/421	0.26 (-4.13, 4.64)
	Trimester 1	1-2 mos	40/624	0.93 (-6.23, 8.09)	10/156	-2.25 (-15.11, 10.61)	52/420	1.19 (-2.58, 4.97)
		3 mos	101/624	2.97 (-1.78, 7.73)	17/156	0.64 (-9.30, 10.59)	35/420	0.29 (-4.22, 4.79)
	Trimester 2	1-2 mos	48/608	-3.68 (-10.28, 2.92)	13/154	2.28 (-9.05, 13.60)	56/409	2.04 (-1.60, 5.68)
		3 mos	108/608	2.38 (-2.25, 7.02)	16/154	-2.81 (-13.11, 7.48)	43/409	0.71 (-3.37, 4.78)
	Trimester 3	1-2 mos	46/618	-3.86 (-10.58, 2.85)	11/146	-3.82 (-16.02, 8.37)	55/417	-0.26 (-3.95, 3.43)
		3 mos	94/618	3.33 (-1.57, 8.23)	19/146	-3.72 (-13.47, 6.02)	35/417	0.38 (-4.11, 4.88)
	Pregnancy	1-5 mos	96/571	-3.58 (-8.47, 1.31)	23/140	1.19 (-7.70, 10.08)	105/369	0.16 (-2.71, 3.02)
		6-9 mos	95/571	2.48 (-2.42, 7.38)	16/140	-2.89 (-13.24, 7.45)	35/369	0.21 (-4.31, 4.72)
Flea/Tick Collar Use	Pre-pregnancy	1-2 mos	1/737	-	5/177	-	2/500	-
		3 mos	29/737	-	0/177	-	12/500	-
	Trimester 1	1-2 mos	3/738	-	1/177	-	4/498	-
		3 mos	26/738	-	4/177	-	12/498	-
	Trimester 2	1-2 mos	9/736	-12.61 (-27.25, 2.03)	1/177	-	2/495	-
		3 mos	23/736	-6.05 (-15.29, 3.18)	4/177	-	15/495	-
	Trimester 3	1-2 mos	6/732	-4.95 (-22.77, 12.87)	1/170	-	1/498	-
		3 mos	22/732	-5.17 (-14.66, 4.32)	4/170	-	12/498	-
	Pregnancy	1-5 mos	14/726	-6.37 (-18.14, 5.39)	2/174	-	11/490	-7.46 (-15.34, 0.41)
		6-9 mos	23/726	-6.27 (-15.52, 2.97)	4/174	-	11/490	0.49 (-7.30, 8.28)
Flea/Tick Skin application	Pre-pregnancy	1-2 mos	27/635	-4.72 (-13.42, 3.98)	4/160	-	24/425	1.47 (-3.97, 6.92)
		3 mos	82/635	0.13 (-5.06, 5.31)	14/160	-	51/425	2.64 (-1.24, 6.53)
	Trimester 1	1-2 mos	29/627	-5.60 (-14.00, 2.79)	2/161	-	27/420	-2.19 (-7.39, 3.01)
		3 mos	91/627	0.87 (-4.10, 5.84)	15/161	-	52/420	3.84 (-0.01, 7.69)
	Trimester 2	1-2 mos	33/624	-2.71 (-10.64, 5.22)	1/161	-	25/426	-2.78 (-8.17, 2.60)
		3 mos	89/624	2.82 (-2.19, 7.83)	15/161	-	49/426	3.23 (-0.70, 7.16)
	Trimester 3	1-2 mos	30/625	-5.43 (-13.64, 2.79)	2/156	-	34/422	2.43 (-2.23, 7.09)
		3 mos	84/625	1.62 (-3.50, 6.75)	11/156	-	43/422	2.93 (-1.22, 7.08)
	Pregnancy	1-5 mos	51/606	-4.60 (-11.07, 1.87)	5/156	-13.78 (-31.31, 3.75)	46/405	-0.13 (-4.22, 3.96)
		6-9 mos	88/606	1.90 (-3.13, 6.94)	13/156	-10.94 (-22.20, 0.33)	49/405	3.91 (-0.03, 7.86)
Flea/Tick Soap, Shampoo, or Powder	Pre-pregnancy	1-2 mos	12/683	-10.30 (-23.01, 2.41)	8/161	-8.81 (-22.90, 5.28)	14/465	-0.64 (-7.58, 6.29)
		3 mos	59/683	-3.97 (-9.91, 1.97)	12/161	-3.76 (-15.34, 7.82)	18/465	-3.29 (-9.43, 2.85)
	Trimester 1	1-2 mos	12/684	-9.85 (-22.61, 2.90)	5/163	-3.77 (-21.52, 13.98)	14/466	-1.75 (-8.68, 5.18)
		3 mos	57/684	-3.78 (-9.82, 2.25)	13/163	0.70 (-10.53, 11.92)	16/466	-3.60 (-10.12, 2.91)
	Trimester 2	1-2 mos	19/682	-9.08 (-19.20, 1.05)	5/166	-2.16 (-19.81, 15.49)	10/469	-3.57 (-11.75, 4.61)
		3 mos	54/682	-5.51 (-11.69, 0.66)	10/166	0.01 (-12.85, 12.87)	19/469	-3.36 (-9.37, 2.64)
	Trimester 3	1-2 mos	15/676	**-13.55 (-24.91, -2.18)**	5/159	0.89 (-16.93, 18.71)	17/465	-3.80 (-10.12, 2.52)
		3 mos	54/676	-4.69 (-10.88, 1.50)	9/159	-1.44 (-15.01, 12.13)	13/465	-2.09 (-9.31, 5.13)
	Pregnancy	1-5 mos	31/663	-1.83 (-9.84, 6.18)	9/160	0.65 (-12.73, 14.03)	23/458	-1.90 (-7.41, 3.60)
		6-9 mos	54/663	-5.64 (-11.82, 0.54)	10/160	0.13 (-12.80, 13.06)	16/458	-3.62 (-10.17, 2.93)
Any Flea/Tick Control Product	Pre-pregnancy	1-2 mos	32/621	-5.07 (-12.99, 2.85)	5/157	1.96 (-15.37, 19.29)	28/421	1.29 (-3.71, 6.29)
		3 mos	130/621	-2.76 (-6.98, 1.47)	23/157	-8.34 (-16.95, 0.27)	71/421	0.30 (-3.02, 3.62)
	Trimester 1	1-2 mos	37/610	-4.09 (-11.52, 3.34)	4/155	-14.11 (-33.28, 5.05)	32/415	-3.91 (-8.65, 0.83)
		3 mos	136/610	-1.44 (-5.61, 2.73)	26/155	-4.75 (-12.89, 3.39)	73/415	1.93 (-1.34, 5.21)
	Trimester 2	1-2 mos	44/606	-3.58 (-10.40, 3.24)	5/157	-0.65 (-17.86, 16.57)	25/422	-3.24 (-8.57, 2.09)
		3 mos	133/606	-1.37 (-5.58, 2.84)	23/157	-7.44 (-16.08, 1.20)	73/422	0.83 (-2.45, 4.10)

Trimester 3	1-2 mos	42/615	-5.44 (-12.38, 1.50)	6/160	-3.08 (-19.05, 12.88)	39/420	0.33 (-4.00, 4.67)
	3 mos	126/615	-1.77 (-6.06, 2.52)	19/160	-6.50 (-15.92, 2.93)	61/420	1.77 (-1.75, 5.29)
Pregnancy	1-5 mos	60/580	-3.22 (-9.13, 2.69)	7/152	-0.08 (-15.05, 14.88)	56/388	-0.82 (-4.53, 2.89)
	6-9 mos	28/580	2.94 (-5.54, 11.43)	7/152	-0.06 (-14.76, 14.65)	13/388	-0.55 (-7.76, 6.67)

Bold typeface indicates statistical significance at $P < 0.05$. Abbreviations: ASD, autism spectrum disorder; DD, developmental delay; DQ, developmental quotient; TD, typically developing; VABS, Vineland Adaptive Behavior Scales

Supplemental Table S10. Beta (95% CI) for associations of frequency of each insecticide with VABS Composite DQ**.**

Application	Exposure Period	Frequency	ASD N Exp/unexp	ASD Beta (95% CI)	DD N Exp/unexp	DD Beta (95% CI)	TD N Exp/unexp	TD Beta (95% CI)
Indoor Professionally Applied Insecticide	Pre-pregnancy	1-2 mos	11/736	2.84 (-6.48, 12.15)	5/176	-	7/498	-6.71 (-18.97, 5.54)
		3 mos	24/736	**6.77 (0.40, 13.14)**	1/176	-	5/498	0.88 (-13.49, 15.24)
	Trimester 1	1-2 mos	8/731	3.28 (-7.61, 14.17)	5/176	-	6/498	-11.45 (-24.60, 1.69)
		3 mos	26/731	5.84 (-0.32, 11.99)	1/176	-	5/498	0.85 (-13.50, 15.21)
	Trimester 2	1-2 mos	19/719	3.43 (-3.68, 10.54)	4/177	-	10/494	-5.09 (-15.38, 5.21)
		3 mos	26/719	2.26 (-3.91, 8.44)	1/177	-	6/494	5.10 (-8.04, 18.23)
	Trimester 3	1-2 mos	16/717	**8.74 (1.03, 16.46)**	1/173	-	11/492	-0.85 (-10.71, 9.01)
		3 mos	26/717	2.81 (-3.34, 8.96)	1/173	-	5/492	0.94 (-13.48, 15.35)
	Pregnancy	1-5 mos	27/705	**7.37 (1.39, 13.35)**	5/176	-	13/490	-0.05 (-9.10, 9.01)
		6-9 mos	28/705	3.19 (-2.74, 9.12)	1/176	-	6/490	-1.38 (-14.57, 11.81)
Indoor Non-professionally Applied Insecticide	Pre-pregnancy	1-2 mos	35/667	-1.00 (-6.37, 4.36)	12/152	-1.83 (-12.67, 9.02)	30/448	2.89 (-3.18, 8.95)
		3 mos	68/667	1.42 (-2.54, 5.37)	15/152	-4.05 (-14.08, 5.97)	33/448	3.09 (-2.66, 8.84)
	Trimester 1	1-2 mos	41/658	1.14 (-3.81, 6.08)	13/147	**11.19 (1.07, 21.31)**	31/448	-0.07 (-6.06, 5.92)
		3 mos	71/658	1.24 (-2.62, 5.11)	19/147	-5.92 (-14.73, 2.90)	32/448	0.07 (-5.80, 5.94)
	Trimester 2	1-2 mos	54/643	0.33 (-4.05, 4.71)	19/145	7.69 (-0.97, 16.35)	42/437	2.81 (-2.38, 8.00)
		3 mos	72/643	-0.03 (-3.88, 3.83)	15/145	-6.08 (-15.85, 3.69)	32/437	2.58 (-3.28, 8.45)
	Trimester 3	1-2 mos	37/661	-1.67 (-6.86, 3.53)	11/144	1.05 (-10.25, 12.35)	27/450	1.57 (-4.89, 8.03)
		3 mos	66/661	0.07 (-3.94, 4.08)	19/144	-4.86 (-13.87, 4.15)	32/450	3.78 (-2.09, 9.64)
	Pregnancy	1-5 mos	106/598	0.35 (-2.95, 3.64)	32/130	1.61 (-5.49, 8.70)	78/403	1.54 (-2.49, 5.57)
		6-9 mos	63/598	0.33 (-3.77, 4.43)	17/130	-3.16 (-12.51, 6.19)	29/403	2.33 (-3.84, 8.50)
Any Indoor Insecticide	Pre-pregnancy	1-2 mos	44/644	-0.36 (-5.16, 4.44)	15/148	-0.64 (-10.28, 9.01)	35/441	1.37 (-4.30, 7.04)
		3 mos	88/644	3.20 (-0.32, 6.72)	16/148	-3.70 (-13.41, 6.02)	37/441	2.47 (-3.01, 7.95)
	Trimester 1	1-2 mos	44/638	1.73 (-3.05, 6.51)	17/143	**9.33 (0.34, 18.32)**	36/441	-2.08 (-7.68, 3.51)
		3 mos	93/638	2.86 (-0.58, 6.30)	20/143	-5.61 (-14.20, 2.98)	36/441	0.44 (-5.13, 6.01)
	Trimester 2	1-2 mos	66/615	1.94 (-2.05, 5.93)	22/141	7.26 (-0.94, 15.46)	46/429	2.04 (-2.96, 7.04)
		3 mos	94/615	0.91 (-2.54, 4.36)	16/141	-5.60 (-15.07, 3.88)	38/429	2.10 (-3.33, 7.53)
	Trimester 3	1-2 mos	48/634	1.87 (-2.73, 6.47)	12/142	2.44 (-8.27, 13.16)	34/440	-0.01 (-5.79, 5.77)
		3 mos	87/634	1.45 (-2.10, 5.01)	20/142	-4.54 (-13.31, 4.24)	37/440	3.42 (-2.07, 8.91)
	Pregnancy	1-5 mos	122/567	2.65 (-0.45, 5.74)	35/129	2.19 (-4.60, 8.98)	82/396	1.29 (-2.66, 5.24)
		6-9 mos	86/567	1.82 (-1.76, 5.41)	18/129	-2.75 (-11.78, 6.29)	35/396	1.77 (-3.89, 7.44)
Outdoor Non-professionally Applied Insecticide	Pre-pregnancy	1-2 mos	32/694	-2.30 (-7.89, 3.29)	6/172	2.07 (-12.74, 16.88)	34/459	**6.26 (0.46, 12.07)**
		3 mos	42/694	0.24 (-4.67, 5.15)	5/172	2.39 (-13.65, 18.44)	14/459	2.36 (-6.46, 11.19)
	Trimester 1	1-2 mos	35/685	0.35 (-5.02, 5.72)	9/167	3.54 (-8.70, 15.77)	38/457	0.71 (-4.80, 6.21)
		3 mos	48/685	0.44 (-4.17, 5.04)	7/167	3.83 (-9.87, 17.54)	12/457	0.13 (-9.45, 9.71)
	Trimester 2	1-2 mos	41/674	-3.77 (-8.74, 1.21)	12/165	**11.00 (0.37, 21.63)**	40/450	3.23 (-2.11, 8.57)
		3 mos	52/674	1.60 (-2.83, 6.03)	6/165	-5.21 (-19.90, 9.47)	18/450	4.14 (-3.68, 11.95)
	Trimester 3	1-2 mos	38/682	-2.18 (-7.36, 2.99)	11/156	-1.77 (-13.04, 9.51)	33/458	-0.62 (-6.52, 5.28)
		3 mos	41/682	1.46 (-3.51, 6.42)	9/156	-1.52 (-13.93, 10.89)	16/458	6.28 (-1.98, 14.54)
	Pregnancy	1-5 mos	89/636	**-4.52 (-8.05, -0.99)**	21/151	4.12 (-4.42, 12.67)	85/413	2.09 (-1.83, 6.00)
		6-9 mos	40/636	2.12 (-2.89, 7.13)	7/151	-5.04 (-18.87, 8.79)	11/413	-0.12 (-10.03, 9.80)

Any Outdoor Insecticide	Pre-pregnancy	1-2 mos	42/639	-1.86 (-6.81, 3.10)	8/161	0.73 (-12.12, 13.58)	49/421	4.01 (-0.91, 8.94)
		3 mos	87/639	3.42 (-0.15, 6.99)	14/161	4.24 (-5.84, 14.32)	37/421	-1.35 (-6.90, 4.19)
	Trimester 1	1-2 mos	40/629	-0.46 (-5.53, 4.60)	10/156	0.14 (-11.62, 11.90)	52/420	1.75 (-3.03, 6.52)
		3 mos	99/629	2.44 (-0.95, 5.83)	17/156	5.09 (-4.01, 14.18)	35/420	-1.02 (-6.72, 4.69)
	Trimester 2	1-2 mos	48/613	-2.67 (-7.34, 2.00)	13/154	8.42 (-1.91, 18.76)	56/409	3.41 (-1.20, 8.02)
		3 mos	106/613	2.26 (-1.04, 5.57)	16/154	1.56 (-7.83, 10.94)	43/409	1.82 (-3.34, 6.98)
	Trimester 3	1-2 mos	46/623	-1.00 (-5.76, 3.76)	11/146	-3.55 (-14.75, 7.64)	55/417	0.31 (-4.36, 4.98)
		3 mos	92/623	2.57 (-0.92, 6.07)	19/146	2.02 (-6.92, 10.96)	35/417	4.26 (-1.43, 9.95)
	Pregnancy	1-5 mos	96/576	-3.39 (-6.84, 0.07)	23/140	3.07 (-5.11, 11.26)	105/369	3.11 (-0.51, 6.72)
		6-9 mos	93/576	2.28 (-1.21, 5.78)	16/140	1.15 (-8.38, 10.68)	35/369	-0.58 (-6.27, 5.12)
Flea/Tick Collar Use	Pre-pregnancy	1-2 mos	1/739	-	5/177	-	2/500	-
		3 mos	29/739	-	0/177	-	12/500	-
	Trimester 1	1-2 mos	3/740	-	1/177	-	4/498	-
		3 mos	26/740	-	4/177	-	12/498	-
	Trimester 2	1-2 mos	9/738	-2.20 (-12.48, 8.08)	1/177	-	2/495	-
		3 mos	23/738	-5.11 (-11.60, 1.37)	4/177	-	15/495	-
	Trimester 3	1-2 mos	6/734	-5.49 (-17.97, 6.99)	1/170	-	1/498	-
		3 mos	22/734	-4.36 (-11.00, 2.29)	4/170	-	12/498	-
	Pregnancy	1-5 mos	14/728	-3.08 (-11.34, 5.18)	2/174	-	11/490	6.07 (-3.88, 16.02)
		6-9 mos	23/728	-5.28 (-11.77, 1.21)	4/174	-	11/490	0.87 (-8.98, 10.71)
Flea/Tick Skin application	Pre-pregnancy	1-2 mos	27/638	-1.37 (-7.44, 4.71)	4/160	-	24/425	2.96 (-3.83, 9.76)
		3 mos	81/638	1.83 (-1.81, 5.47)	14/160	-	51/425	3.13 (-1.72, 7.97)
	Trimester 1	1-2 mos	30/629	-0.73 (-6.51, 5.06)	2/161	-	27/420	-2.14 (-8.67, 4.38)
		3 mos	90/629	2.18 (-1.31, 5.67)	15/161	-	52/420	2.35 (-2.47, 7.17)
	Trimester 2	1-2 mos	33/627	-0.53 (-6.07, 5.01)	1/161	-	25/426	-2.32 (-9.06, 4.43)
		3 mos	88/627	2.74 (-0.78, 6.25)	15/161	-	49/426	2.16 (-2.76, 7.07)
	Trimester 3	1-2 mos	30/628	-0.13 (-5.87, 5.60)	2/156	-	34/422	-0.09 (-5.95, 5.77)
		3 mos	83/628	2.80 (-0.79, 6.39)	11/156	-	43/422	2.26 (-2.95, 7.47)
	Pregnancy	1-5 mos	52/608	-1.34 (-5.83, 3.15)	5/156	-	46/405	-1.21 (-6.34, 3.93)
		6-9 mos	87/608	2.54 (-1.00, 6.08)	13/156	-	49/405	2.81 (-2.14, 7.76)
Flea/Tick Soap, Shampoo, or Powder	Pre-pregnancy	1-2 mos	12/685	-2.71 (-11.62, 6.20)	8/161	-11.28 (-24.05, 1.49)	14/465	2.92 (-5.89, 11.73)
		3 mos	60/685	-2.73 (-6.86, 1.41)	12/161	-7.64 (-18.12, 2.85)	18/465	-2.06 (-9.87, 5.75)
	Trimester 1	1-2 mos	12/686	0.39 (-8.54, 9.33)	5/163	-9.42 (-25.56, 6.71)	14/466	2.72 (-6.05, 11.50)
		3 mos	58/686	-2.73 (-6.93, 1.48)	13/163	-5.18 (-15.37, 5.02)	16/466	-4.62 (-12.87, 3.64)
	Trimester 2	1-2 mos	19/684	-4.24 (-11.33, 2.86)	5/166	-11.21 (-27.23, 4.82)	10/469	-0.95 (-11.27, 9.38)
		3 mos	55/684	-2.91 (-7.20, 1.38)	10/166	-4.77 (-16.43, 6.90)	19/469	-1.92 (-9.52, 5.67)
	Trimester 3	1-2 mos	15/678	-3.73 (-11.72, 4.25)	5/159	-7.65 (-23.86, 8.56)	17/465	1.10 (-6.91, 9.11)
		3 mos	55/678	-2.90 (-7.22, 1.42)	9/159	-6.39 (-18.73, 5.94)	13/465	-3.16 (-12.31, 5.98)
	Pregnancy	1-5 mos	31/665	-0.61 (-6.24, 5.02)	9/160	-7.99 (-20.13, 4.15)	23/458	4.38 (-2.56, 11.32)
		6-9 mos	55/665	-2.94 (-7.24, 1.36)	10/160	-4.88 (-16.61, 6.85)	16/458	-4.45 (-12.71, 3.81)
Any Flea/Tick Control Product	Pre-pregnancy	1-2 mos	32/624	-2.05 (-7.64, 3.55)	5/157	-3.91 (-19.67, 11.86)	28/421	2.99 (-3.29, 9.27)
		3 mos	130/624	-0.91 (-3.90, 2.07)	23/157	**-10.38 (-18.22, -2.54)**	71/421	0.83 (-3.34, 5.00)
	Trimester 1	1-2 mos	38/612	-0.55 (-5.74, 4.64)	4/155	-9.27 (-26.76, 8.22)	32/415	-0.32 (-6.30, 5.67)
		3 mos	136/612	-0.11 (-3.05, 2.83)	26/155	**-8.23 (-15.65, -0.80)**	73/415	0.97 (-3.17, 5.10)
	Trimester 2	1-2 mos	44/609	-1.09 (-5.90, 3.73)	5/157	-6.81 (-22.55, 8.92)	25/422	-1.29 (-8.00, 5.42)
		3 mos	133/609	-0.18 (-3.15, 2.79)	23/157	**-8.06 (-15.96, -0.17)**	73/422	1.03 (-3.09, 5.15)

Trimester 3	1-2 mos	42/618	-2.17 (-7.07, 2.73)	6/160	-5.93 (-20.49, 8.63)	39/420	-0.97 (-6.41, 4.48)
	3 mos	126/618	-0.18 (-3.21, 2.84)	19/160	**-8.63 (-17.22, -0.03)**	61/420	1.44 (-2.98, 5.86)
Pregnancy	1-5 mos	61/582	-2.27 (-6.40, 1.86)	7/152	-5.67 (-19.31, 7.97)	56/388	1.91 (-2.77, 6.58)
	6-9 mos	28/582	3.67 (-2.30, 9.63)	7/152	-2.57 (-15.97, 10.84)	13/388	-2.96 (-12.03, 6.12)

Bold typeface indicates statistical significance at $P< 0.05$. Abbreviations: ASD, autism spectrum disorder; DD, developmental delay; DQ, developmental quotient; TD, typically developing; VABS, Vineland Adaptive Behavior Scales

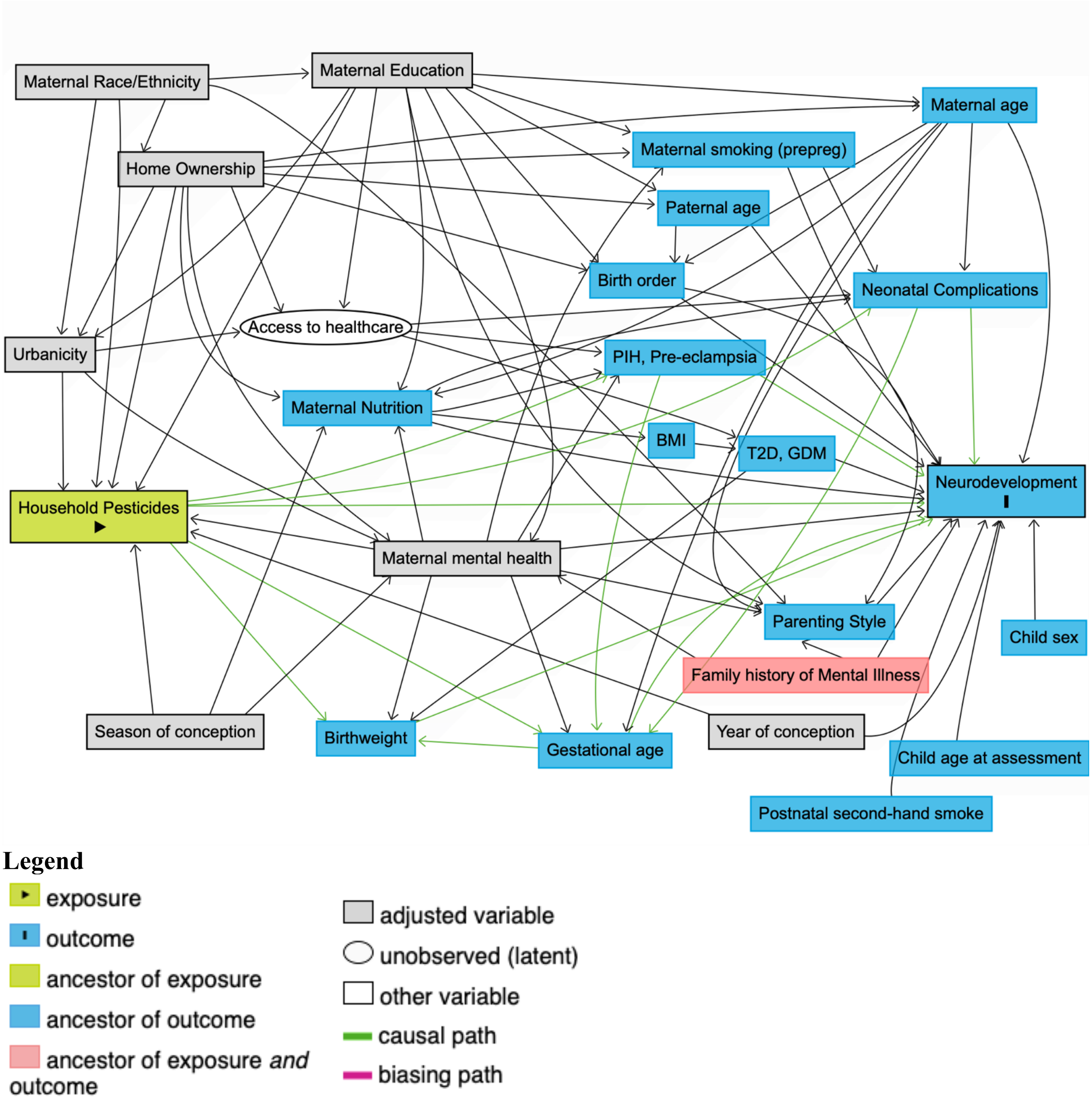

Supplemental Figure S1. Directed acyclic graph (DAG) for the association of prenatal exposure to household insecticides and autism spectrum disorder (ASD), in the CHARGE study population. Figure generated using DAGitty[4]. Variables in grey were selected by DAGitty as a minimally sufficient adjustment set and all but season of conception was included in the final multivariable regression model. Abbreviations: BMI, body mass index; GDM, gestational diabetes; T2D, type II diabetes,
